# Therapeutic role of extracellular vesicles from human umbilical cord mesenchymal stem cells and their wide therapeutic implications in inflammatory bowel disease and other inflammatory disorder

**DOI:** 10.3389/fmed.2024.1406547

**Published:** 2024-07-30

**Authors:** Muhammad Azhar Ud Din, Aijun Wan, Ying Chu, Jing Zhou, Yongmin Yan, Zhiliang Xu

**Affiliations:** ^1^Changzhou Key Laboratory of Molecular Diagnostics and Precision Cancer Medicine, Wujin Hospital Affiliated with Jiangsu University, Jiangsu University, Changzhou, China; ^2^Key Laboratory of Medical Science and Laboratory Medicine of Jiangsu Province, School of Medicine Jiangsu University, Zhenjiang, China; ^3^Zhenjiang College, Zhenjiang, China

**Keywords:** inflammatory bowel disease, human umbilical mesenchymal stem cells, exosomes, genes, extracellular vesicles

## Abstract

The chronic immune-mediated inflammatory condition known as inflammatory bowel disease (IBD) significantly affects the gastrointestinal system. While the precise etiology of IBD remains elusive, extensive research suggests that a range of pathophysiological pathways and immunopathological mechanisms may significantly contribute as potential factors. Mesenchymal stem cells (MSCs) have shown significant potential in the development of novel therapeutic approaches for various medical conditions. However, some MSCs have been found to exhibit tumorigenic characteristics, which limit their potential for medical treatments. The extracellular vesicles (EVs), paracrine factors play a crucial role in the therapeutic benefits conferred by MSCs. The EVs consist of proteins, microRNAs, and lipids, and are instrumental in facilitating intercellular communication. Due to the ease of maintenance, and decreased immunogenicity, tumorigenicity the EVs have become a new and exciting option for whole cell treatment. This review comprehensively assesses recent preclinical research on human umbilical cord mesenchymal stem cell (hUC-MSC)-derived EVs as a potential IBD therapy. It comprehensively addresses key aspects of various conditions, including diabetes, cancer, dermal injuries, neurological disorders, cardiovascular issues, liver and kidney diseases, and bone-related afflictions.

## 1 Introduction

Inflammatory bowel disease (IBD) refers to a group of chronic inflammatory disorders of the gastrointestinal tract, with two primary subtypes: Crohn’s disease (CD) and ulcerative colitis (UC) (1). These disorders result from an abnormal immune response in genetically susceptible individuals, triggered by environmental factors. The characteristic symptoms include abdominal pain, diarrhea, rectal bleeding, weight loss, and fatigue (2). The chronic and relapsing nature of IBD often leads to a diminished quality of life and a higher risk of complications such as bowel strictures, abscesses, and even colorectal cancer. Over the years, various treatment modalities have been developed to treat patient with IBD including anti-inflammatory medications, immunosuppressant, biological therapies, and surgical interventions (3). While these treatments have been effective for many patients, they are not without limitations. Long-term use of immunosuppressive drugs can lead to increased susceptibility to infections, and biologic therapies often come with a high financial burden. Moreover, a significant proportion of IBD patients do not respond adequately to existing treatments, highlighting the urgent need for novel therapeutic strategies (4).

The complexities of IBD, including its multifactorial etiology and heterogeneity in disease presentation, pose a challenge for clinicians seeking to tailor treatment approaches to individual patients (5). Currently, a significant proportion of the etiology and pathology underlying this condition remains elusive to the scientific community. Nevertheless, it is widely acknowledged that the condition is characterized by a polygenic and multifactorial nature (6). Incorporating the insights obtained from numerous recent studies, the fundamental elements contributing to the onset of IBD involve genetic interactions, dysregulated mucosal immune responses prompted by environmental factors, and disruptions in the regulation of the gut microbiota (6). Currently, several treatment protocols, including immunomodulatory, thiopurine agents, and monoclonal antibodies targeting tumor necrosis factor (anti-TNF), are employed for the management of IBD. However, these treatments have been found to lack the attainment of sufficiently favorable therapeutic outcomes (7). Consequently, researchers are actively exploring the development of advanced clinical techniques and strategies for the treatment of IBD. In this context, the search for safer and more effective therapies has led researchers to explore the regenerative potential of mesenchymal stem cells (MSCs) and their extracellular vesicles (EVs) (8, 9). MSCs are multipotent, adult stem cells found in various tissues, including bone marrow, adipose tissue, and the umbilical cord (10). These cells have garnered immense interest in the field of regenerative medicine due to their remarkable self-renewal capabilities and their ability to differentiate into multiple cell lineages, including osteocytes, adipocytes, and chondrocytes (11, 12). Moreover, the quantity of stem cells and their capacity for proliferation and differentiation exhibited a marked decline with advancing age (13), thereby imposing limitations on the application of these cells in clinical trials (14). The morphological characteristics, immunophenotype, proliferation rate, multi-directional differentiation capacity, and their potential to induce hematopoietic stem cell (HSC) differentiation in umbilical cord-derived mesenchymal stem cells (UC-MSCs) closely resemble those observed in bone marrow-derived mesenchymal stem cells (BM-MSCs) (15), but, it is noteworthy that UC-MSCs display a heightened proliferative capacity and lower levels of human leukocyte antigen (HLA)-ABC and HLA-DR expression in comparison to BM-MSCs (16). Furthermore, it is worth noting that UC-MSCs exhibit a diverse array of stem cell types, making them readily available and easily collectable resource that can be efficiently preserved (17, 18). Consequently, UC-MSCs assume a distinctive role in diminishing both the frequency and severity of graft-versus-host disease (GVHD), a complication that affects over 50% of patients undergoing hematopoietic stem cell transplantation (HSCT) (18). Moreover, owing to their inherent migratory potential towards cancer cells, numerous studies have suggested the utilization of UC-MSCs in cell-based therapies aimed at targeting tumors and facilitating the localized delivery of anti-cancer agents (19). Nonetheless, several facets of research concerning UC-MSCs are still in their early stages of development. UC-MSCs are considered optimal candidate cells for cell replacement therapy, primarily due to their minimal immunogenicity, robust proliferative potential, and capacity for differentiation (20).

The therapeutic effectiveness of MSCs do not exclusively hinge on their ability to differentiate into various cell types. Instead, their paracrine effects, which involve the release of trophic factors and EVs, assume a central role in promoting tissue repair and modulating the immune response (21). MSCs can orchestrate a coordinated response by influencing local cell populations, reducing inflammation, and promoting tissue regeneration (22). The secretion of EVs, in particular, has gained significant attention for their role in intercellular communication and their potential as therapeutic agents (23). EVs are small membranous vesicles released by virtually all cell types, including MSCs. They are involved in cell-to-cell communication and serve as vehicles for the transfer of bioactive molecules, including proteins, lipids, and nucleic acids, between cells (24, 25). EVs are classified into several subtypes, including exosomes, macrovesicles, and apoptotic bodies, based on their biogenesis and size. Among these, exosomes, typically ranging from 30 to 150 nanometers in diameter, have garnered significant interest for the therapeutic potential (26). Exosomes are released into the extracellular space through the fusion of multivesicular bodies with the plasma membrane (27). Extensive investigations have revealed the secretion of exosomes by a wide range of cell types, including mast cells, dendritic cells (27), B cells (28), T cells (29), tumor cells (30), and epithelial cells. Exosomes have also been found in numerous kinds of body fluids, such as saliva, urine, breast milk, and plasma (31, 32) urine (33, 34). Exosomes, like their parent cells, carry a cargo of bioactive molecules that can modulate various cellular processes (35, 36). These molecules include growth factors, cytokines, microRNAs, and lipids, all of which can influence recipient cells’ behavior (37, 38). This cargo is carefully packaged within the exosome’s lipid bilayer, protecting it from degradation and ensuring its efficient delivery to target cells (39). This unique characteristic makes exosomes ideal candidates for therapeutic interventions, as they can harness the regenerative power of their parent cells in a more controlled and targeted manner (40, 41). Exosomes originating from diverse sources exert an influence on the etiology of IBD (42). The significance of intercellular communication in maintaining homeostasis in multicellular organisms has been well-documented previously. Consequently, exosomes, which are secreted by a majority of cells, play a critical role in forming a network and actively participating in intracellular signaling (43). They facilitate the transfer of bioactive components including, lipids, nucleic acids, and proteins from one cell to another, thereby initiating biological responses in the recipient cells (44). Exosomes have demonstrated a greater efficacy than their parent cells and can be stored without compromising their functionality, making them an appealing focus of research. Recently, there has been an increasing interest in utilizing exosome administration as a novel therapeutic strategy to expedite preclinical research endeavors (45, 46). Furthermore, exosome-delivered miRNAs contribute to lymphangiogenesis and play a role in IBD. Exosomes derived from adipose tissue-derived MSCs modulate the miRNA-132/TGF-β pathway, thereby promoting VEGF-C-dependent lymphangiogenesis (47). MSC-derived exosomes have been substantiated to augment angiogenesis in endothelial cells by transporting miR-125a (48). Conversely, BM-MSCs promote lymphangiogenesis through the secretion of VEGF-A, which stimulates lymphatic endothelial cells (LECs) to activate the VEGFR-2 pathway (49).

The therapeutic potential of EVs, including those derived from MSCs, extends beyond their immunomodulatory effects (50, 51). Studies have demonstrated that MSC-derived EVs can promote tissue repair and regeneration in various disease models, including myocardial infarction, stroke, and cartilage injury (52). The cargo carried by these vesicles plays a pivotal role in modulating the recipient cell’s behavior, promoting angiogenesis, reducing fibrosis, and enhancing tissue remodeling (52). The isolation and characterization of human umbilical cord mesenchymal stem cell (hUC-MSC)-derived EVs represent a critical step in harnessing their therapeutic potential (53). Researchers have developed various methods to isolate and purify these vesicles, including ultracentrifugation, size exclusion chromatography, and immunoaffinity-based techniques (54).

These methods ensure the enrichment of exosomes and other EV subtypes from hUC-MSC culture supernatants, allowing for their subsequent analysis and utilization (55). One of the most striking features of hUC-MSC-derived EVs is their ability to modulate the immune response (56). These vesicles can suppress the activation of pro-inflammatory immune cells while promoting the expansion of regulatory T cells and M2 macrophages, thus shifting the immune milieu towards an anti-inflammatory and tissue-healing phenotype (57). This immunomodulatory capacity has profound implications for the treatment of immune-mediated disorders like IBD (58). Recent preclinical studies and early-phase clinical trials have provided compelling evidence of the therapeutic potential of hUC-MSC-derived EVs in the management of IBD (59). These studies have shown that the administration of hUC-MSC-derived EVs can ameliorate disease symptoms, reduce inflammation, and promote mucosal healing in animal models and human patients with IBD. The mechanisms underlying these effects involve the immunomodulatory properties of the vesicles, as well as their ability to enhance epithelial barrier function and promote tissue repair (60, 61). The therapeutic potential of hUC-MSC-derived EVs extends beyond gastrointestinal disorders. Researchers are exploring their use in various neurological disorders, such as Parkinson’s disease, Alzheimer’s disease, and spinal cord injury (SCI) (62, 63). These vesicles have shown promise in promoting neuroprotection, reducing inflammation, and enhancing neural tissue repair in preclinical models (64). Cardiovascular diseases, including myocardial infarction and heart failure, are leading causes of morbidity and mortality worldwide. The hUC-MSC-derived EVs have emerged as potential candidates for cardiac regeneration and repair. Their ability to stimulate angiogenesis, reduce oxidative stress, and modulate immune responses has made them attractive for the treatment of cardiovascular disorders (65, 66). Musculoskeletal disorders, such as osteoarthritis and bone fractures, present significant challenges in the field of regenerative medicine. The hUC-MSC-derived EVs have shown promise in promoting bone and cartilage regeneration by enhancing the proliferation and differentiation of osteoblasts and chondrocytes. These vesicles may offer a minimally invasive and cell-free alternative to traditional treatments (67).

While the therapeutic potential of hUC-MSC-derived EVs is promising, several challenges must be addressed before their widespread clinical adoption. Standardization of isolation and characterization methods, determination of optimal dosing regimens, and long-term safety assessments are crucial steps in the path to clinical translation (54). Moreover, regulatory approvals and manufacturing scalability need to be addressed to ensure the accessibility of these therapies to a broader patient population. The heterogeneity of diseases like IBD underscores the importance of personalized medicine. Identifying biomarkers that can predict patient responses to hUC-MSC-derived EV therapy is a critical research area. Biomarker discovery will enable clinicians to select the most appropriate patients for treatment and tailor therapy regimens accordingly, maximizing therapeutic outcomes (68). As the field of regenerative medicine advances, ethical and regulatory considerations become increasingly important (69). Ensuring the ethical sourcing of umbilical cord tissue and transparent reporting of research findings is essential. Regulatory agencies must also develop clear guidelines to govern the production and clinical use of hUC-MSC-derived EVs, balancing innovation with patient safety (61). The therapeutic role of hUC-MSC-derived EVs represent a promising frontier in the treatment of IBD and a wide array of other disorders (70). With their ability to modulate the immune response, promote tissue repair, and enhance regenerative processes, hUC-MSC-derived EVs hold the potential to revolutionize the way we approach the management of chronic and debilitating conditions (71).

This study has undertaken an exploration of the complexities surrounding IBD, delving into the regenerative capabilities of MSCs, revealing the therapeutic potential inherent in EVs, and engaging in a discussion concerning the promising prospects of hUC-MSC-EVs within the context of IBD. The journey towards fully harnessing the therapeutic potential of these minuscule communicators is an ongoing endeavor, replete with a myriad of challenges and opportunities that await both researchers and clinicians. As we continue to venture deeper into the realm of regenerative medicine, we stand at the precipice of uncovering innovative solutions that have the potential to transform the lives of individuals grappling with chronic and presently incurable diseases. This review underscores the paramount importance of sustained research and clinical progress within this promising field, emphasizing the potential of hUC-MSC-EVs as an exceptional therapeutic avenue not only for IBD but also for a spectrum of inflammatory conditions.

## 2 Extracellular vesicles

The release of EVs is a fundamental biological process in both prokaryotic and eukaryotic cells, occurring under normal physiological conditions as well as in aberrant situations. Despite being written off in the past as little more than biological waste with little significance, recent studies have highlighted their crucial function as bioactive transporters. These vesicles mediate a wide range of biological processes and act as transporters of many cellular components, enabling complex cellular communications (72). Proteins such as cell surface receptors, signaling proteins, transcription factors, enzymes, and extracellular matrix proteins are among the diverse cargo carried by EVs (73). Additionally, they have lipids and nucleic acids (DNA, mRNA, and miRNA) that can be transferred from donor to recipient cells to facilitate molecular transfer and intercellular communication (74). It has been discovered that EVs are linked to pathological conditions like cancer, heart disease, and neurological illnesses (75). EVs comprise a range of subtypes that are categorized based on their mechanisms of synthesis and release, such as exosomes, apoptotic blebs, and other EV subgroups (76). Additionally, they can be categorized according to the type of cell from which they originated, such as endothelium or platelet-derived cells, or according to the physiological state of the cells, such as “prostasomes” coming from the prostate and “oncosomes” originating from cancer cells. The primary components of EVs include apoptotic bodies, exosomes, and microvesicles (77). However, other forms have been discovered recently, including membrane particles, large oncosomes, migrasomes (78), ectosomes (78), exomeres (79) and supermeres (Table 1).

**Table 1:** Extracellular vesicles as therapeutic tools and targets for diseases.

Subtype	Size	Origin of EV	Density	Biomarkers	Mechanism	References
Exosomes	50–150	Multivesicle body	1.13–1.19	CD9, CD63, CD81, Tsg101, ALIX, HSP70	Endosomes develop into late endosomes, which have intraluminal vesicles that fuse with the plasma membrane to release MVBs (dependent or independent of ESCRT)	(80, 81)
Microvesicles	100–1,000	Plasma membrane	1.032–1.068	Integrins, Selectins, CD40, tissue factor	Direct plasma membrane budding and cleavage are caused by calcium influx and remodelling of the cortical cytoskeleton	(81, 82)
Migrasomes	500–3,000	Retraction fibers	Unknown	Tspan4, CD63, Annexin A1	Actin polarisation and cell migration cause migratory cells to migrate and create migratory granules at the tip or by bifurcating the retraction fibres	(83)
Apoptotic Bodies	100–5,000	Plasma membrane	1.16–1.28	Annexin V, C3b, thrombospondin, Annexin A1, histone coagulation factor	Programed cell death involves the fragmentation of cytoplasm	(81, 84, 85)
Exomeres	Secreting from cells	≤50	1.1–1.19	TGFBI, ENO1 and GPC1	Large cytoplasmic extensions are cleaved off the cell body	(86)
Oncosomes	1,000–10,000	The shedding of non-apoptotic plasma membrane blebbing	1.10–1.15	Cav-1 or ADP ribosylation factor 6	Released by cancerous cells with amoeboid movement	(87, 88)
Supermeres	∼35 (<50)	Unknown	Unknown	TGFBI, ACE2, PCSK9, miR-1246, MET, GPC1 and AGO2, exRNA; miR-1246	Unknown	(89, 90)

Recently, EVs have emerged as a promising therapeutic option for a range of diseases and conditions, including BD (91). In animal models of IBD, studies have provided evidence showing that EVs derived from various sources, including MSCs, possess the ability to mitigate inflammation and facilitate tissue healing (92). Using EVs as a therapeutic alternative presents several challenges that need to be addressed. In the preparation of EVs, standardization of isolation and characterization is a significant challenge in maintaining the purity and quality of EVs (93). The immunogenicity potential and pro-coagulant effects are two additional safety concerns associated with EV-based medicines (94). Despite these obstacles, EV-based medicines are still being clinically researched and developed, demonstrating significant potential for the treatment of various inflammatory illnesses including IBD. Herein, the MSC-derived-exosomes are the main focus of the study to provide comprehensive use, specifically in the treatment of IDB and other pathological inflammatory conditions in general.

## 3 Exosomes

Exosomes, a type of EVs, which originate from endosomes, typically exhibit an average diameter of approximately 50–100 nm (95). The plasma membrane undergoes a sequential process of invagination, leading to the eventual formation of multivesicular bodies (MVBs). These MVBs have the ability to interact with other intracellular vesicles and organelles, thereby contributing to the diversity of components found in exosomes. Depending on the specific cell they originate from, exosomes, can encompass a wide range of cellular constituents, such as DNA, RNA, lipids, metabolites, as well as cytosolic and cell-surface proteins (96).

Exosomes, in particular, have emerged as crucial mediators of cellular communication, playing significant roles in both normal physiological processes, such as lactation (97), immune response (34) and neuronal function (97), and also in the development and progression of diseases, such as liver disease (97), neurodegenerative diseases (98) and cancer. Exosomes are increasingly recognized as promising therapeutic agents for gastrointestinal conditions, IBD, using a cell-free therapeutic strategy (98, 99).

Numerous diseases associated with IBD, characterized by disruptions in mucosal immune responses, compromised intestinal barrier integrity, and alterations in the balance of intestinal microbial populations, are orchestrated through pathways involving exosomal intercellular communication (100). Exosomes, being complex molecules, are discharged into human serum and other bodily fluids, and their functional contents exhibit variations between IBD patients and healthy individuals (101). Consequently, exosomes may have the potential to serve as diagnostic biomarkers that reflect the current state of IBD (101, 102).

### 3.1 Characteristics and properties of exosomes

#### 3.1.1 Characteristics of exosomes

Exosomes originate from endosomal vesicles within the cell and exhibit distinctive characteristics that set them apart from other cell-secreted microvesicles (103). They are enclosed by a phospholipid bilayer architecture, with a size ranging from 30 to 150 nanometers and a density falling within the range of 1.13 to 1.19 grams per milliliter (104, 105). When observed through transmission electron microscopy (TEM), exosomes present a cup-shaped morphology, further confirming their identity. Moreover, the presence of specific proteins, including tetraspanins (e.g., CD63, CD9, and CD81) and β-actin, serves as additional markers to differentiate exosomes from other vesicular structures (101, 104). To emphasize their prevalence, it is noteworthy that approximately 1 × 10^12^ exosomes can be found in just 1 milliliter of blood (105). These distinct characteristics and abundance make exosomes a subject of significant interest in various fields of research and clinical applications (106).

#### 3.1.2 Biogenesis of exosomes

Exosome biogenesis is a highly regulated process that occurs in various cell types under both pathological and physiological conditions. The secretion of exosomes is governed by the modulation of Rab27a and Rab27b expression (107). A diverse array of cell types, including lymphocytes, dendritic cells, fibroblasts, erythrocytes, platelets, mast cells, tumor cells, stem cells, monocytes, macrophages, natural killer (NK) cells, B lymphocytes, and T lymphocytes, are known to synthesize exosomes (108).

**Figure 1:**
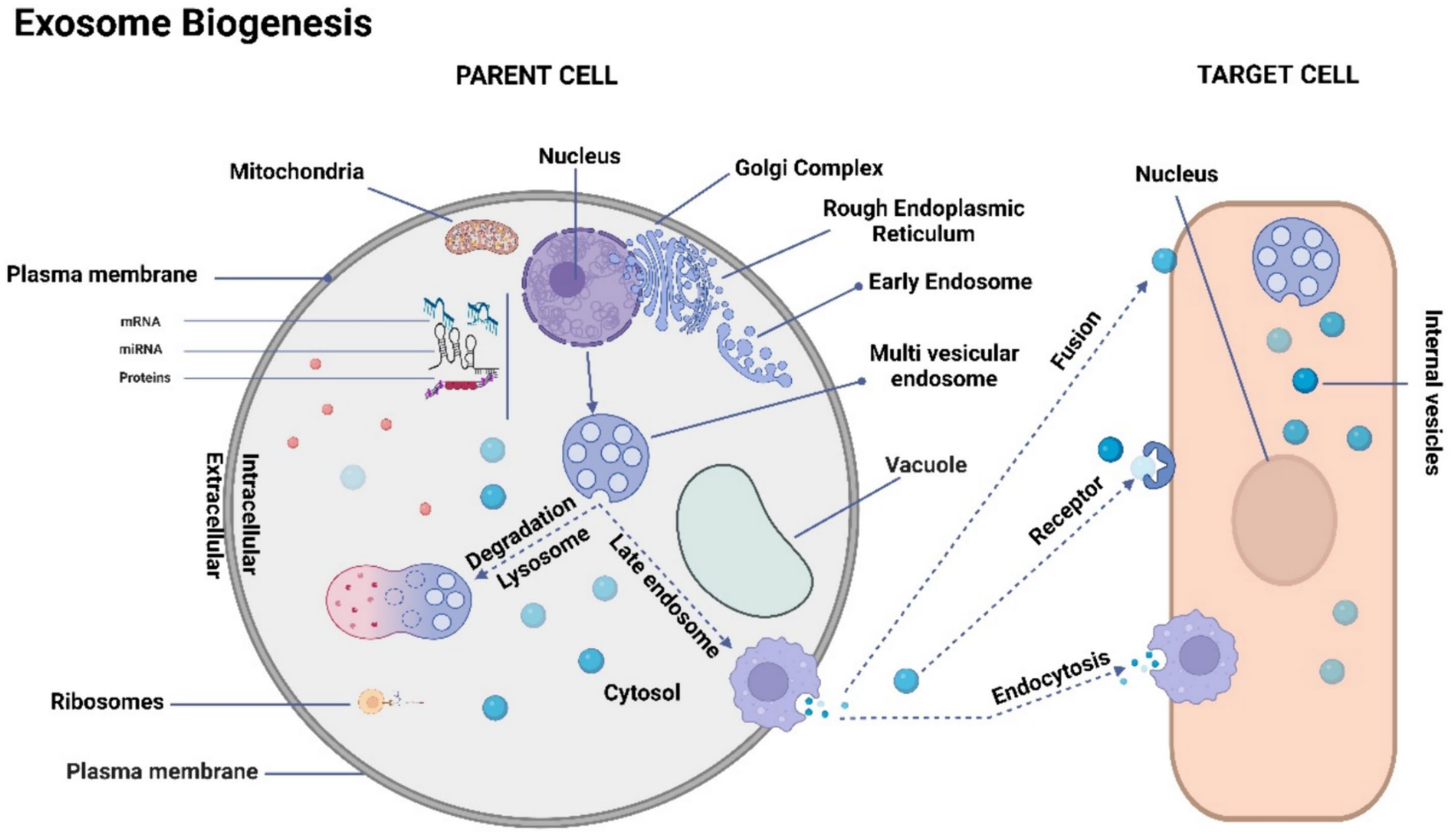
Schematic representation of exosome biogenesis. The process of exosome biogenesis is illustrated schematically in this figure. During exosome biogenesis, multivesicular endosomes (MVEs) initially undergo invagination, resulting in the formation of small intracellular vesicles. These vesicles encapsulate a diverse array of cytoplasmic cargo molecules, including proteins, messenger RNAs (mRNAs), and microRNAs (miRNAs). Notably, MVEs demonstrate two distinct fusion pathways: one involves fusion with the cellular membrane, facilitating the exocytosis of the enclosed exosomes (referred to as intravesicular vesicles). The other pathway entails fusion with lysosomes, leading to the degradation of the contents within MVEs. Consequently, exosomes gain entry into recipient cells through two distinct mechanisms: initially, they traverse the endocytic route, followed by internalization by the recipient cell. Secondly, they can merge directly with the recipient cell’s plasma membrane, subsequently releasing their cargo into the cytoplasm. It’s important to note that cells possess the capacity to generate membrane-derived vesicles that bud directly from the plasma membrane. These vesicles serve as vehicles for transporting functional proteins, RNAs, and other bioactive molecules.

The process of exosome biogenesis commences with the internalization of the cell membrane, leading to the formation of small intracellular structures referred to as early endosomes (103–107). Early endosome development involves a progressive maturation process that culminates in the generation of intraluminal vesicles (ILVs). On the other hand, late endosomes, known as multivesicular bodies (MVBs), are formed through the inward folding of segments of the endosomal membrane (106). The fusion of MVBs with the plasma membrane results in the exocytotic release of ILVs into the extracellular space, where they ultimately undergo transformation into exosomes (99–102, 105). This intricate process is visually depicted in Figure 1.

Notably, exosomes are present in a variety of physiological fluids, including serum, as well as in saliva, amniotic fluid, and breast milk (106). Their presence in these fluids underscores the ubiquity of exosomes in biological systems and their potential significance in various biological and clinical contexts.

#### 3.1.3 Composition of exosomes

**Figure 2:**
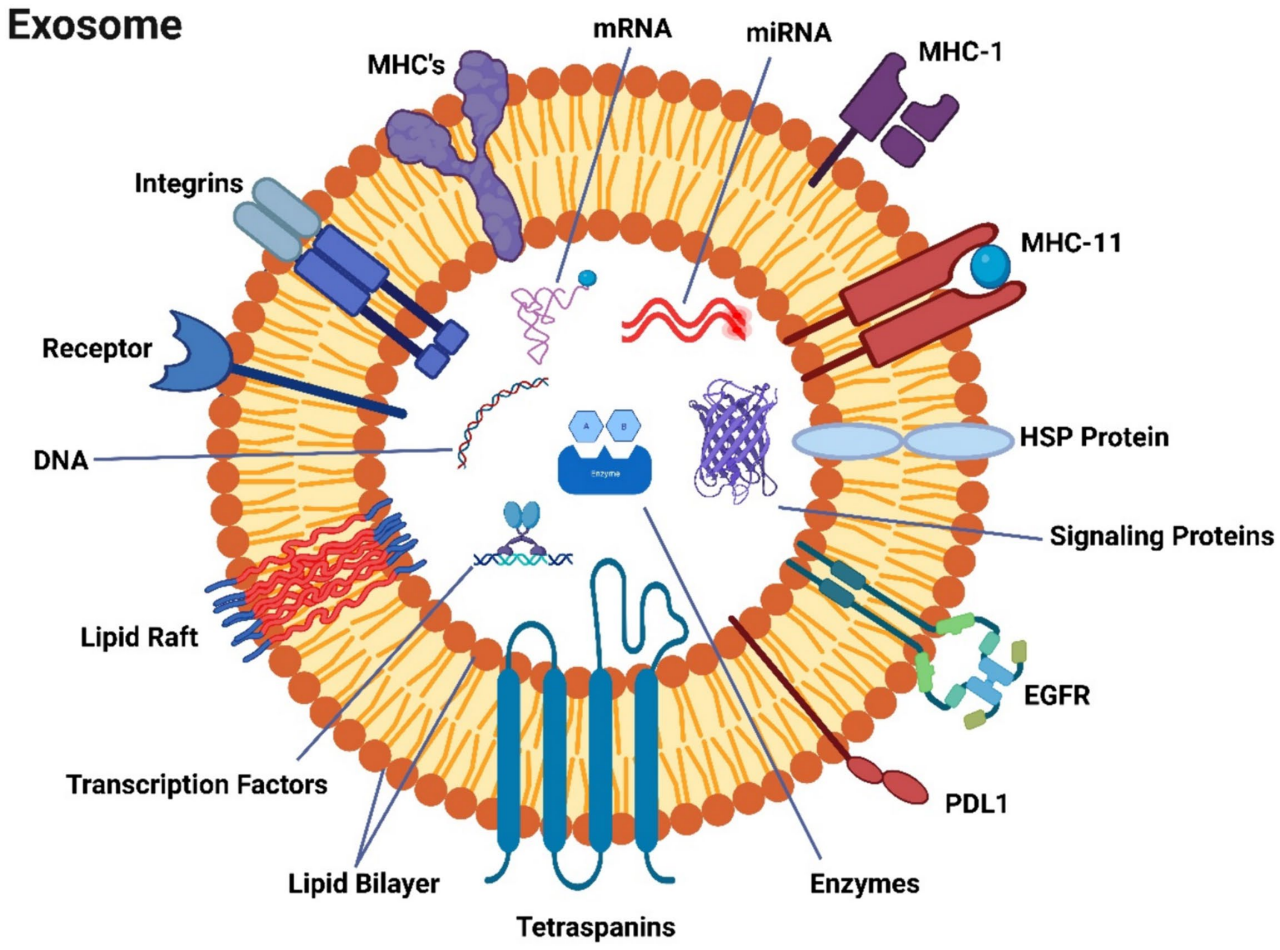
A description of exosomes’ molecular make-up.

Exosomes, which are minuscule in size and invisible to the naked eye and standard light microscopes, can only be observed using electron microscopy. Their morphology is characterized by flattened spheres, which can result from the dehydration process required for electron microscopy preparation, leading to their collapse (108, 109). Exosomes are complex structures composed of proteins, particularly those derived from the plasma or endosomal membrane, lipids, and various cytosolic components (Figure 2). It’s important to note that exosomes lack proteins originating from the Golgi apparatus, nuclear pore complex, mitochondria, and endoplasmic reticulum (Figure 2). Exosomes lack proteins of the Golgi apparatus, nuclear pore complex, mitochondria and endoplasmic reticulum (110). Data on exosomal content are systematically curated and updated in databases such as ExoCarta, Vesiclepedia, and EVpedia (111). Although initial investigations suggest the presence of proteins from the cytosol, endosomes, and plasma membrane in exosomes, it is essential to acknowledge that cellular organelles such as mitochondria, the Golgi apparatus, or the nucleus do not exclusively consist of proteins. The establishment of exosomal protein databases has been a collaborative effort among multiple research teams. Exosome repositories like ExoCarta^1^ and Vesiclepedia^2^ have cataloged a total of 9,769 proteins, 1,116 lipids, 3,408 mRNAs, and 2,838 miRNAs within exosomes originating from various cellular sources and organisms up to the present day (110). Exosome protein sorting, a newly explored field of study, relies, at least in part, on the endosomal sorting complex required for transport (ESCRT) machinery and protein ubiquitylation (112). During exosome biogenesis, ESCRT orchestrates the formation of intricate structures on the plasma membrane, resembling membranous necks. These structures encompass conical funnels, tubular arrangements, planar spirals, and filaments, which are hypothesized to regulate membrane remodeling processes.

Exosomes encompass a substantial number of transport proteins, including tubulin, actin, and actin-binding molecules (110), in addition to various proteins intricately involved in specific roles within secretory cells (113). Exosomal proteins are categorized into diverse groups based on their family, function, and subcellular localization (114). The most frequently encountered protein categories in exosomes include those related to (i) the formation of multivesicular bodies (MVBs), (ii) transmembrane proteins acting as targeting or adhesion molecules, such as tetraspanins like CD9, CD63, and CD81, which play roles in membrane fusion, (iii) signal transduction proteins such as annexin and 14-3-3 proteins, (iv) cytoskeletal proteins including actin, syntenin, and myosin, (v) chaperones like HSPA8 and HSP90, and (vi) metabolic enzymes, such as GAPDH, LDHA, PGK1, aldolase, and PKM (111, 113). Each individual exosome also contains MHC class I molecules, among distinct components (114), and heat shock proteins (112). These proteins participate in antigen presentation and antigenic peptide attachment to MHC class I molecules (113). Tetraspanin family members CD9, CD63, CD81, and CD82 interact with other transmembrane proteins to facilitate antigen presentation and adhesion. The interaction of CD9 and CD82 with integrins can inhibit the migration and invasion of tumor cells (111). Exosomes exhibit distinctive lipid compositions in addition to their protein content (113). Intriguingly, they are deficient in lysobisphosphatidic acid and intraluminal vesicle (ILV) lipids (115) but contain high levels of sphingomyelin, phosphatidylserine, ceramide, and cholesterol.

Exosomes are recognized for their substantial content of DNA and RNA, alongside lipids and proteins. The term “EV-DNA” refers to DNA enclosed within EVs, spanning a size range of 100 base pairs to 2.5 kilobases (115). Analysis of complete RNA sequencing data from EVs isolated from serum suggests that RNA repeats constitute approximately 50% of the total EV-RNA content. Additionally, miRNAs and tRNAs are estimated to account for up to 15% of the EV-RNA content (115, 116). Certain RNAs are found in higher concentrations in exosomes than in the originating cells, particularly in exosomes from MSCs (116). Numerous studies have provided evidence that RNA can indeed be transferred between cells via exosomes (117). However, the extent to which transferred RNA retains its functionality in recipient cells, as well as the proportion that undergoes fragmentation and subsequent transport, remains an area of ongoing investigation.

## 4 Human umbilical cord mesenchymal stem cells origin and biological characteristics

**Figure 3:**
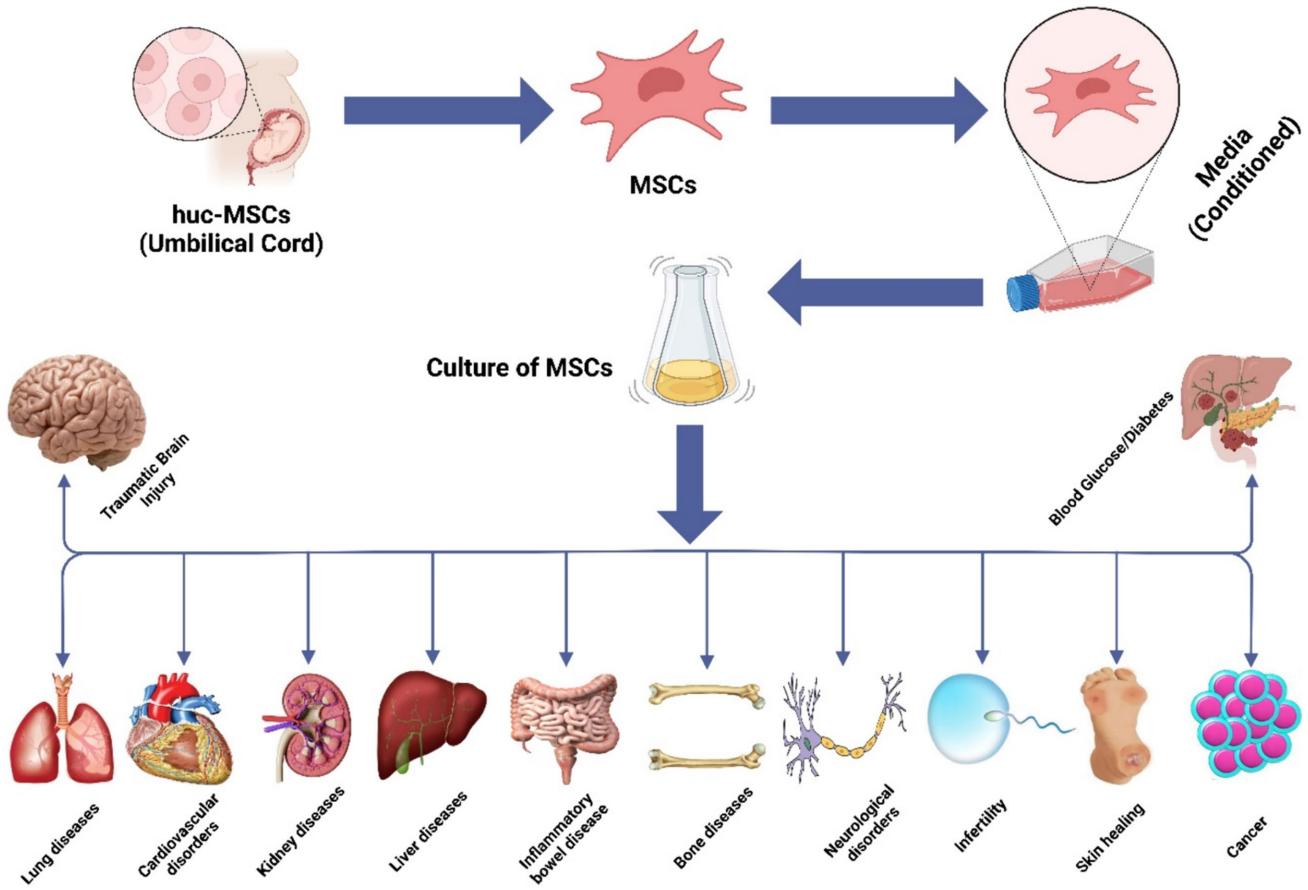
Illustration of the hUC-MSC in various abnormalities.

MSCs have gained significant prominence in experimental cell-based therapeutic approaches for a variety of human ailments. They are widely employed due to their proven efficacy in numerous disease-related animal models and their excellent safety record in clinical settings. MSCs offer great potential for treating human illnesses because of their ability to differentiate, self-renew, and modulate the immune response (118). While MSCs have garnered attention as a potential cellular treatment for various medical conditions, emerging evidence suggests that their therapeutic benefits are primarily mediated through EVs produced via paracrine processes (119). These EVs play a crucial role in conveying the therapeutic advantages of MSCs. The hUC-MSCs have been the subject of numerous research projects that have explored their potential use for the treatments of various medical problems, including diabetes (120), cancer (121), liver (122), bone (123), cartilage (124), brain (125), cardiovascular issues (126), and IBD (127), and Figure 3 illustrates the therapeutic effects of hUC-MSC, both in *in vivo* and *in vitro* environments. The administration of hUC-MSCs demonstrates marked amelioration in pivotal parameters, encompassing the disease activity index, fluctuations in body weight, alterations in colonic length, and histopathological assessments of colitis, predominantly through the mitigation of inflammation. A discernible correlation exists between the dosage of hUC-MSCs and the extent of therapeutic response as these parameters serve as standard indicators in IBD evaluations, reflecting both disease severity and the effectiveness of therapeutic interventions (126–128).

### 4.1 Biological characteristics

#### 4.1.1 The UC-MSC’s (hUC-MSC) immunological characteristics

The subcortical endothelium of the umbilical cord, Wharton’s jelly (WJ), and the perivascular area are the primary locations where UC-MSCs are commonly found. Wharton’s Jelly has a structure primarily resembling a sponge, with collagen fibers, proteoglycans, and stromal cells intertwined within it (20). When analyzing UC-MSCs from Wharton’s Jelly using flow cytometry, it was discovered that CD24 and CD108 were abundantly expressed, while the dermal fibroblast marker CD40 and fibroblast-specific markers (FAP and FSP) were not discernible, which highlights the abundance of MSCs in the Wharton’s Jelly region (128, 129). UC-MSCs can exhibit distinctive markers corresponding to various cell lineages, exemplifying their pluripotent nature (130). In contrast to hematopoietic stem cells, UC-MSCs exhibit the expression of MSC-specific markers such as CD105, CD90, and CD73, along with adhesion molecule markers including CD54, CD13, CD29, and CD44. Conversely, UC-MSCs shows reduced or absent expression of surface antigens CD31, CD14, CD34, and CD45 (131). Additionally, these cells are deficient in immune response-related antigens necessary for T lymphocyte activation, including CD80, CD86, CD40, and CD40L, as well as the MHC class II antigen HLA-DR. (132) UC-MSCs exhibit reduced immunogenicity compared to BM-derived cells due to their considerably lower expression levels of CD106 and HLA-ABC (133).

#### 4.1.2 UC-MSCs’ capability to proliferate and differentiate

WJ-MSCs, which exhibited the highest proliferation rate, outperformed adipose tissue and BM-MSCs by a factor of three to four in terms of proliferation (134). Based on the inquiry conducted by the Mennan research team, no significant disparity in the rate of proliferation was observed. The population of UC-MSCs exhibited an average twofold increase between passages P0 and P3 within a span of 2–3 days. This rate of expansion significantly exceeds the duration observed for BM-MSCs (135). UC-MSCs have the capacity for multidirectional differentiation, enabling them to differentiate into various tissues such as bone, fat, cartilage, and others (136). Therefore, in the field of regenerative medicine, these cells are considered ideal seed cells due to their potential to facilitate the healing of various tissues and organs (137). According to studies, chemokines are produced by biological tissues that undergo damage due to ischemia-anoxia or persistent inflammation (138). These chemokines aid in recruiting MSCs to the injury site and subsequently regulate their migration to facilitate their differentiation into various cell lineages (139). Under specific conditions in an *in vitro* setting, UC-MSCs have been observed to exhibit the ability to undergo “trans-differentiation,” resulting in their differentiation into osteoblast-like mesodermal cell types (140), endothelial cells (141), cardiomyocytes (142), as well as ectoderm-derived hepatocytes with potential for neuronal transformation (143), and pancreatic cells (144), bridging the germinal layers within the endoderm.

#### 4.1.3 hUC-MSC-EVs (exosomes) isolation and characterization

To maximize the therapeutic effects of EVs, it is essential for researchers to isolate and characterize them. The isolation and characterization of hUC-MSC-EVs hold great importance for their potential therapeutic use in IBD and other associated illnesses (145). However, the extraction of EVs from biological fluids, such as serum or conditioned media, can be challenging due to the heterogeneity of EV populations and the presence of impurities (93). In response to this challenge, various methodologies have been developed to achieve optimal purity and cost-effectiveness of EVs. These techniques include ultracentrifugation, density gradient centrifugation, and size-exclusion chromatography, all aiming to achieve high levels of purity (146). Ultracentrifugation, are commonly used technique for EV extraction, involves differential centrifugation at high speeds, reaching up to 100,000 x g, to pellet EVs (147). This method can be further optimized by utilizing sucrose or iodixanol gradients to separate EVs from other subcellular components (148). Another technique, density gradient centrifugation, employs a continuous gradient to separate EVs based on their buoyant density. This method is often used for isolating specific EV subpopulations, such as exosomes (149). Size-exclusion chromatography (SEC) is another method used for EV isolation, where EVs are separated based on their size characteristics. This technology is useful for removing unwanted contaminants, such as proteins and lipoproteins, which may be present during the isolation process (150). Once the isolation process is complete, hUC-MSC-EVs can be characterized by analyzing their physical and biochemical properties. Physical characterization includes assessing various attributes of EVs, such as their dimensions, structure, and abundance. Techniques such as dynamic light scattering (DLS), transmission electron microscopy (TEM), and nanoparticle tracking analysis (NTA) are commonly employed for this purpose (151). Biochemical characterization involves the detection and analysis of specific EV markers, including CD9, CD63, and CD81, using techniques like western blotting, flow cytometry, or immunogold labeling (152). Furthermore, the cargo of hUC-MSC-EVs can be characterized through proteomics, RNA sequencing, and lipidomics, providing insights into their functional properties and potential therapeutic targets (153).

It is important to acknowledge the inherent challenges associated with the extraction and characterization of EVs, which stem from the diverse nature of EV populations and the potential risk of contamination (154). Therefore, it is crucial to employ a range of isolation and characterization methodologies to ensure the integrity and homogeneity of hUC-MSC-EVs for their potential clinical application. By improving separation and characterization techniques and tailoring the cargo of EVs to therapeutic targets, researchers can develop novel IBD medications that are more effective and exhibit fewer side effects than current treatments. For a comprehensive overview of different techniques for exosome isolation and characterization, please refer to Table 2.

**Table 2:** A comparative analysis of various techniques for isolating exosomes.

Technique used	Principle	Dimension	Advantages	Drawbacks	References
Ultrafiltration	Immune affinity	Large	Convenient, efficient and logical	Impure, exosomes with reduced purity may exhibit a tendency to partially adhere to the cellular membrane	(155)
Immune-affinity capture	Shape and molecular size	Small	Pureness/increased purity	Expensive and yielding is very low	(156)
Ultracentrifugation	Antigen and antibody specific binding	Large	Inexpensive reagent cost, the likelihood of pollution is minimal	Costly apparatus and significant amount of time along with the biological activity and integrity of exosomes are compromised due to their poor quality	(157)
Size exclusion chromatography	Density, molecular size, and shape	Medium	Assurance of exosome yield, purity, integrity, and biological activity can be achieved	Utilization of specialized equipment	(158)
Microfluidic	Molecular size	Small	Affordability, convenience, and automation	Verification of selectivity and specificity is required	(159)

## 5 Role of UC-MSC/hUC-MSC-derived EVs in IBD

MSC-derived EVs have been shown in numerous studies to be an effective treatment for experimentally induced colitis in mice. One study documented and examined the weight loss, stool viscosity, and hematochezia in mice with DSS-induced colitis treated with MSCs, MSC-Exs, and placebo in distinct groups. The findings demonstrated that MSCs and MSC-Exs both had an equal anti-inflammatory impact and could treat colitis in mice (160). Additionally, studies on MSCs’ immediate and long-term protective effects on experimental colitis have shown that MSCs produced from human adipose tissue not only temporarily relieve colitis but also have positive long-term regulatory effects on IBD (161) (Table 3).

**Table 3:** Unveiling the role of MSCs and MSC-Exo in IBD treatment.

Disease	Administration route	Therapy	Model/Sample	Results	References
IBD	Intraperitoneal injection	HucMSC-Exs	*In vivo* (mice)	MSC-Exs reduced IBD in mice by means of TSG-6-mediated mucosal barrier repair and intestinal immunological homeostasis restoration	(162)
IBD	Intraperitoneal injection	cAT-MSCs	*In vivo* (mice)	TSG-6 secreted by cAT-MSCs induced a shift in the macrophage phenotype from M1 to M2 in mice, alleviating IBD symptoms and regulating the expression of pro-and anti-inflammatory cytokines in the colon	(163)
IBD	Intraperitoneal injections	BM-MSCs	*In vivo* (mice)	In the peritoneum, BM-MSCs accumulated and produced the immunomodulatory factor TSG-6, which reduced intestinal inflammation	(164)
UC	Peritoneal injection	BMSC-Exs	*In vivo* (mice)/*in vitro* (LPS-treated macrophages)	BMSC-Exs reduced the inflammatory response by down-regulating pro-inflammatory proteins, up-regulating anti-inflammatory proteins, and promoting the conversion of macrophages into the M2 phenotype	(165)
IBD	Intraperitoneal injection	ADMSC-Exs	*In vivo* (mice)	AdMSC-Exs may alleviate the clinical signs of IBD by modulating Treg populations and cytokines	(166)
IBD	Intraperitoneal injections	MSCs	*In vivo* (mice)	In patients with colitis, hUCMSCs enhanced the proportion of Tr1 cells in the spleen and mesenteric lymph nodes, decreased the percentage of helper T cells (Th1 and Th17 cells), promoted the expansion of Tr1 cells, and inhibited apoptosis, effectively alleviating IBD	(167)
IBD	Intravenous infusion	MSC-Exs (miR-378a-3p)	*In vivo* (mice)/*in vitro* (IEC-6)	MSCs-Exs suppress IBD by reducing GATA2 expression and downregulating AQP4, thereby inhibiting the PPAR signaling pathway	(168)
IBD T	Intravenous infusion	-MSCs	*In vivo* (mice)	Intravenous infusion of T-MSCs increased circulating IGF-1 levels and ameliorated colitis in mice	(169)
IBD	Enemas	MSCs	*In vivo* (mice)	Activating the Nrf2/Keap1/ARE pathway could be an effective strategy for MSCs to promote intestinal mucosal healing in experimental colitis	(170)

### 5.1 Mechanism of MSC-derived EVs in the treatment of IBD

**Figure 4:**
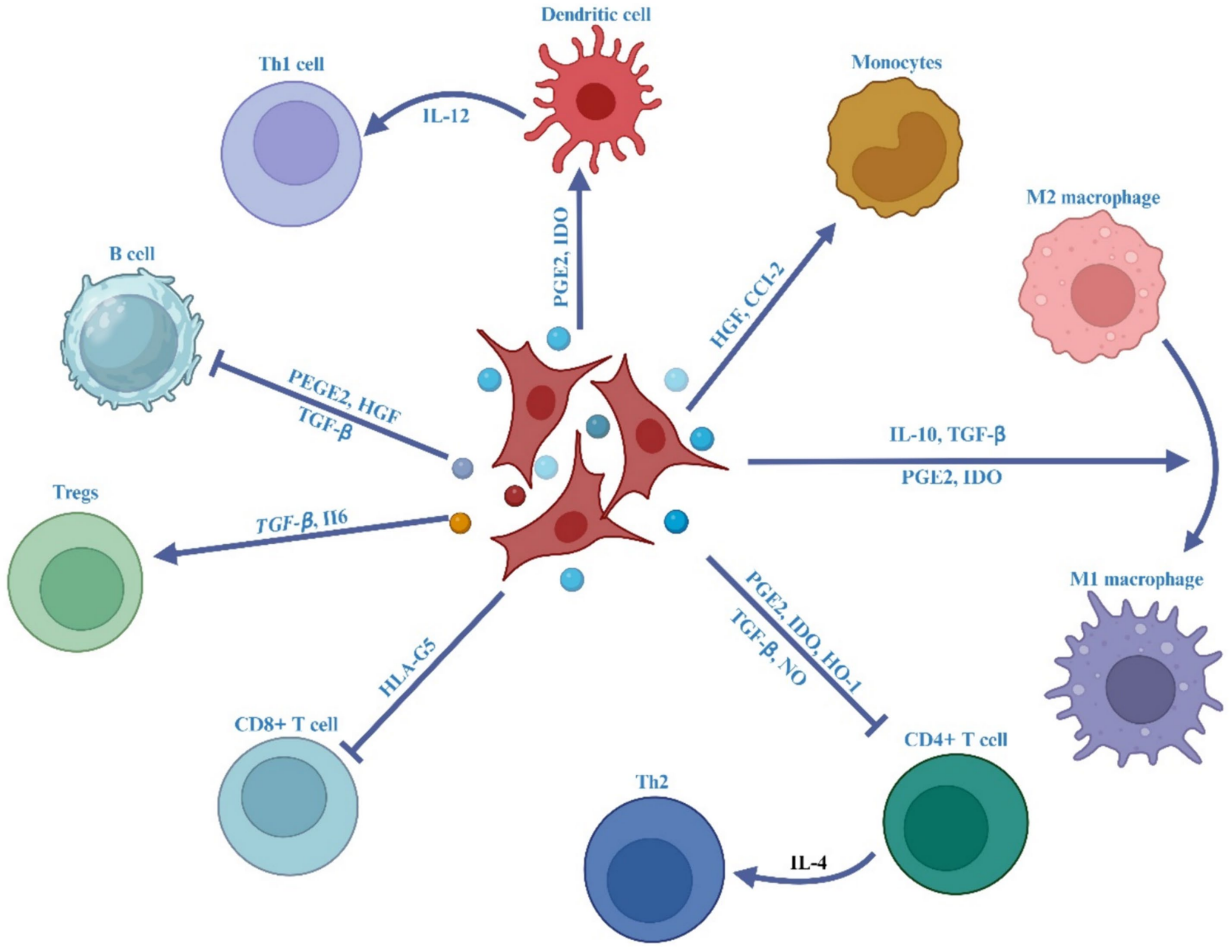
The impact of MSC-EVs on immune effector cells in IBD.

It has been documented that the MSC-Exos have the ability to home in on areas of intestinal inflammation, interact with immune cells like macrophages, T lymphocytes, and DCs, and modulate the characteristics and activities of immune cells by releasing bioactive substances, such as cytokines, to regulate irregular immune responses and suppress inflammatory reactions (153), as outlined in Figure 4.

Furthermore, MSC-Exos possess the capability to influence intestinal epithelial cells (IECs), facilitate the restoration of the intestinal epithelial barrier (IEB), mitigate oxidative stress, and alleviate colon fibrosis. Therefore, exhibit potential in the treatment of IBD (171). The therapeutic utilization of MSC-derived EVs in the treatment of IBD are given in Table 4.

**Table 4:** Therapeutic application of MSC-derived EVs for treating IBD.

Sources	Effector molecules	Mechanism	Effect	References
hUC-MSCs	miRNA-326	NF-κB signaling pathway and enzymes associated to neddylation	Preventing neddylation and reducing colitis	(172)
hUC-MSCs	miRNA-378a-5p	IL-1β, IL-18, the NLRP3 axis, and caspase-1	Reducing colitis by controlling the pyroptosis of macrophages	(173)
hUC-MSCs	miRNA-378a-5p	NLRP3 axis	Suppressing colitis brought on by DSS and controlling macrophage pyroptosis	(174)
AD-MSCs	miRNA-132	TGF-β/Smad signaling and Smad-7	Facilitating lymphangiogenesis reliant on VEGF-C	(175)
hUC-MSCs	miRNA-146a	SUMO1 axis	Preventing colitis	(172)
hUC-MSCs	lnc78583-miRNA-3202	HOXB13 axis	Alleviate bowel inflammation	(176)
BM-MSCs	MiRNA-200b	HMGB3 axis	Reduce the inflammatory damage that IECs have caused	(177)
BM-MSCs	miR-125a miR-125b	Stat3 axis; prevent the development of Th17 cells	Reduce the severity of colitis brought on by DSS	(178)
hUC-MSCs	TSG-6	TJ repair by upregulating TJ protein expression. Th2 and Th17 cell immune responses have been altered	Reestablishing intestinal immunological homeostasis and mucosal barrier restoration	(162)
hBM-MSCs	MT-2	M2b macrophages polarized	Lessen irritation of the mucosa	(179)
AD-MSCs	N/A	Immunomodulatory properties; control the number of Tregs	Reduce inflammation in acute colitis caused by DSS	(166)
Olfactory Ecto-MSCs	N/A	Controls Th1/Th17 subpopulations and differentiation	Reduce the intensity of IBD disease	(180)
hUC-MSCs	N/A	Suppress the production of IL-7 and iNOS	Reduce IBD and relieve inflammatory reactions	(173)
hUC-MSCs	N/A	Control the ubiquitination modification	hUC-MSCs lessen the colitis’s intensity	(181)
BM-MSCs	N/A	Reduce apoptosis, oxidative stress, and intestinal inflammation	Reduce IBD’s intensity	(182)
AD-MSCs	N/A	Process including inflammation, apoptosis, and immunity	Reduce the harm to the intestinal epithelium	(183)

### 5.2 Macrophages

**Figure 5:**
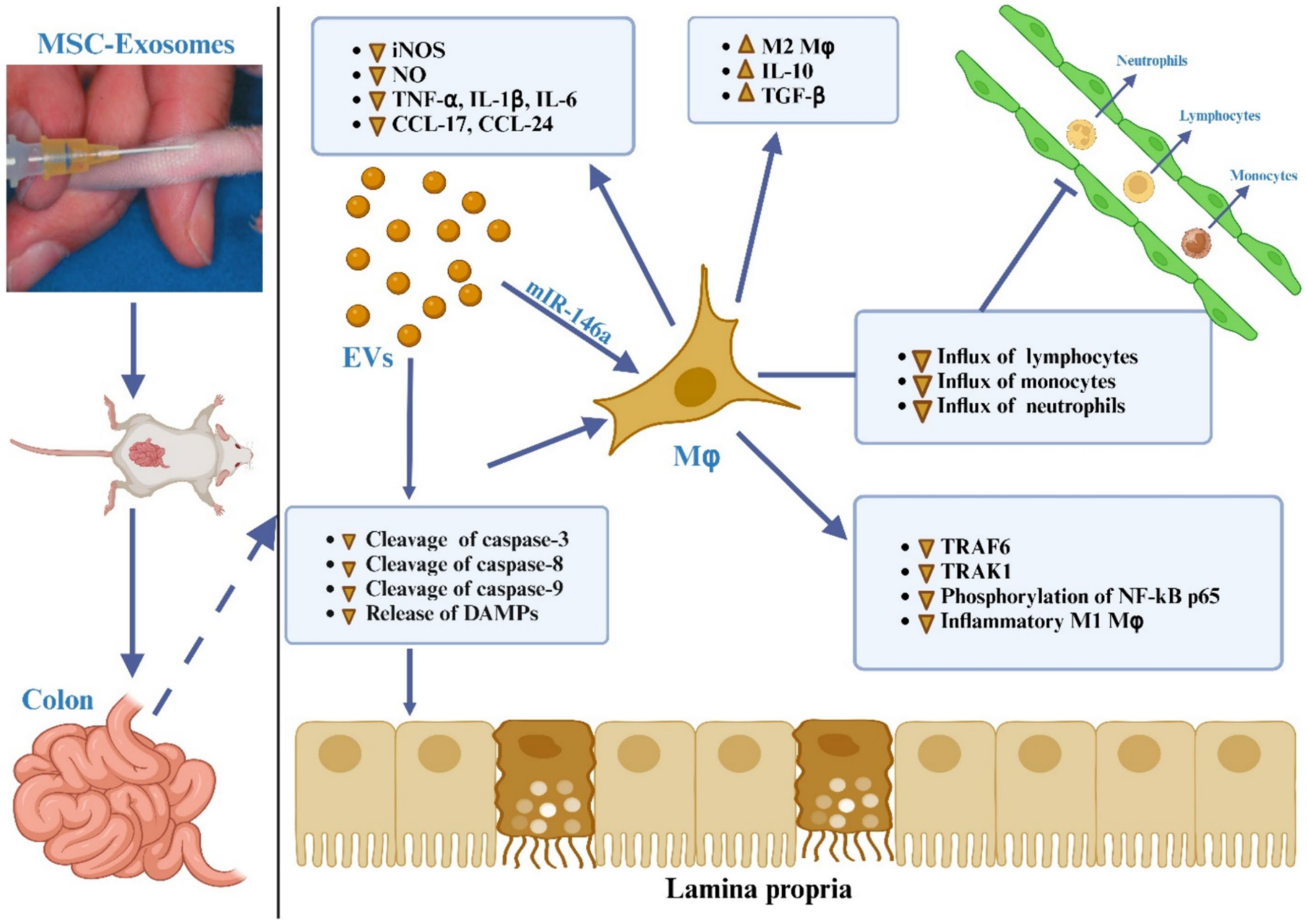
The MSC-EVs mitigate ulcerative colitis is through modulating the phenotype and function of colonic macrophages. MSC-EVs reduce the cleavage of caspase-3, -8, and -9 and lessen the release of damage-associated molecular patterns (DAMPs) from damaged gut epithelial cells, leading to decreased activation of the NF-κB signaling pathway in colon macrophages. MSC-EVs deliver miR-146a, which inhibits the expression of TNF receptor-associated factor 6 (TRAF6) and IL-1 receptor-associated kinase 1 (IRAK1), reduces phosphorylation of NF-κB p65, and suppresses the generation of inflammatory M1 macrophages. This is evidenced by decreased inducible nitric oxide synthase (iNOS) expression, significantly lower production of nitric oxide (NO), inflammatory cytokines (TNF-α, IL-1β, IL-6), and chemokines (CCL-17, CCL-24), resulting in reduced recruitment of neutrophils, monocytes, and lymphocytes to the inflamed gut. Additionally, MSC-EVs promote the polarization of colon macrophages to an anti-inflammatory M2 phenotype, marked by increased secretion of immunosuppressive cytokines TGF-β and IL-10, thus alleviating colitis.

It has been determined that macrophages are the primary cells responsible for causing colon inflammation (184, 185), as depicted in Figure 5. Upon activation by pro-inflammatory stimuli, macrophages are mobilized to inflamed areas and undergo differentiation into macrophages with distinct polarities under the influence of chemokines and inflammatory factors. M1 macrophages release proinflammatory cytokines (IL-1β, IL-6, TNF-α, and IL-12) as well as Th1 chemokines (CXCL9, CXCL10, and CXCL11), which are involved in antigen presentation, T cell activation, and the initiation of an adaptive immune response. M2 macrophages release suppressive cytokines like IL-10 and TGF-β, which serve to dampen immune reactions and counteract inflammatory responses (186, 187). Anomalous polarization of macrophages contributes to immune irregularities within the intestinal mucosa and mediates the onset of intestinal inflammation, a key triggering factor in the development of IBD (188). Research indicates that the regulation of macrophage polarization and the balance between M1 and M2 macrophages is crucial in the immunotherapeutic approach to IBD (187–190). Both laboratory studies and animal trials have demonstrated that when lipopolysaccharide (LPS)-activated macrophages are co-cultured with BM-derived MSC-Exos, fluorescently labeled MSC-Exos are observed within the macrophages after 24 h. This interaction leads to the modulation of macrophage polarization towards the M2 phenotype, resulting in a decreased M1/M2 ratio and reduced expression of IL-6, IL-7, TNF-α, and IL-12, along with diminished macrophage infiltration in colon tissue (165, 173). Additionally, further research has indicated that adipose-derived MSC-Exos harbor an anti-inflammatory agent, TNF-α stimulated gene-6 (TSG-6), plays a pivotal role in orchestrating the polarization of M2 macrophages (191). The potential pathways through which MSC-Exos regulate macrophages include:

#### 5.2.1 Transfer of miRNA

At the site of intestinal inflammation, MSC-Exos bind to macrophages and release encapsulated miRNA, which in turn selectively modulates the mRNA within macrophages and influences the polarization of M2 macrophages. Research has demonstrated that the ExomiRNAs of MSCs, including miR-146a, can downregulate the expression of TNF receptor-related factor 6 (TRAF-6) and IL-1 receptor-related kinase 1 (IRAK-1), thereby suppressing the release of proinflammatory cytokines and promoting the expression of the anti-inflammatory factor IL-10 (192).

#### 5.2.2 Anti-inflammatory proteins delivery

MSC-Exo contains a multitude of proteins, notably metallothionein-2 (MT-2), renowned for its capacity to suppress colitis activity. This protein plays a pivotal role in mitigating the intestinal inflammatory response by upholding the integrity of the intestinal barrier and fostering the polarization of M2b macrophages (179).

#### 5.2.3 Induction of Toll-like receptors

MSC-Exos penetrate macrophages, potentially triggering the myeloid differentiation primary response gene 88 (MyD88)-dependent signaling pathway in macrophages upon recognition of TLR3 by the enclosed dsRNA. This activation leads to the induction of M2 polarization in macrophages, the release of anti-inflammatory factors, and the inhibition of the inflammatory response (193).

#### 5.2.4 Competitive inhibition of CC chemokine receptor 2

In the presence of inflammatory stimuli, the interaction between CC chemokine receptor 2 (CCR2) expressed on the surface of monocytes and CC chemokine ligand 2 expressed on the surface of macrophages triggers the migration of monocytes to inflammatory sites, where they transform into macrophages, thereby intensifying the inflammatory response and exacerbating tissue damage. Notably, MSC-Exos exhibit high expression of CCR2, which competitively binds to the chemokine ligand 2 on the surface of macrophages. This competitive binding inhibits the recruitment and activation of monocytes, prevents the polarization of M1 macrophages, and reduces the expression of proinflammatory cytokines, such as IL-1β, IL-6, and TNF-α, thereby effectively restraining inflammatory responses (194).

#### 5.2.5 Regulation of inflammatory cytokines releases

Firstly, MSC-Exos derived from MSCs, induced by specific inflammatory factors, encapsulate numerous anti-inflammatory cytokines and chemokines. Upon uptake by macrophages, these components are released into the surrounding environment. Secondly, the aforementioned pathways, once activated, govern or initiate the polarization of M2 macrophages, leading to the upregulation of anti-inflammatory cytokines and the downregulation of proinflammatory cytokines. This orchestration serves to sustain the equilibrium of anti-inflammatory factors at inflammatory sites and effectively suppress excessive inflammatory responses (195).

### 5.3 T lymphocytes

The continual exposure of antigens to CD4^+^ T cells by immune cells in the intestinal mucosa prompts the differentiation of primitive CD4^+^ T cells (Th0) into various helper T cell subtypes (predominantly Th1, Th2, and Th17) and regulatory T (Treg) cells, under the influence of antigen presentation and cytokine regulation. The trajectory of their differentiation plays a crucial role in preserving intestinal immune equilibrium and modulating inflammatory responses within the intestine (196). Research indicates that the co-cultivation of T lymphocytes with MSC-Exos results in the downregulation of cyclinD-2 and the upregulation of P27KIP-1, thereby impeding T lymphocytes from entering the S phase and inhibiting their growth and proliferation. This suggests that MSC-Exos have the capacity to modulate the proliferation and differentiation of T lymphocytes. Notably, MSC-Exos primarily regulate the equilibrium between Th1 and Th2, as well as Treg and Th17 cells (196).

#### 5.3.1 The regulation of the transformation balance between Th1 and Th2

Th1 and Th2 cells represent the two primary subtypes of Th0 differentiation. Th1 cells primarily express IFN-γ and IL-12, while Th2 cells predominantly express IL-4 (196, 197). The dysregulation of Th1/Th2 subsets is intricately linked to the chronic inflammation observed in IBD (197, 198). The direction of differentiation is largely influenced by the concentration of IL-12 in the environment and the activation status of antigen-presenting cells. Through the regulation of DCs and macrophages, MSC-Exos induce the polarization of M2 macrophages, impede the antigen presentation of DCs, and diminish the release of proinflammatory cytokines, such as IL-12. This environment is conducive to the transition of T cells into Th2 cells. Studies have demonstrated that MSC-Exos can markedly diminish the expression of IL-12, hinder the differentiation and proliferation of Th1 cells, and facilitate the transition of T cells into Th2 cells following co-cultivation with T cells activated by phytohemagglutinin (199, 200). Furthermore, research has revealed that the TSG-6 protein detected in hUC-MSC-exo regulates the immune response of Th2 and Th17 cells in mesenteric lymph nodes (MLN), downregulates proinflammatory cytokines in colon tissue, and upregulates anti-inflammatory cytokines, thereby safeguarding the integrity of the intestinal barrier (162).

#### 5.3.2 The regulation of the transformation balance between Treg and Th17

Treg cells play a pivotal role in inhibiting the transformation of effector T cells and adaptive mucosal immunity in the intestine, thereby reducing immune-related tissue damage in IBD. A study has revealed that MSC-Exos induce T lymphocyte apoptosis and modulate the differentiation and proliferation of Treg cells through programmed death ligand and galactosin-1 (201). Treg cells express anti-inflammatory cytokines, such as IL-10 and TGF-β, to achieve immunosuppressive effects (193, 202, 203). In contrast, Th17 cells express proinflammatory cytokines that contribute to the inflammatory activity in IBD. Notably, the ratio of Th17/Treg in the peripheral blood of IBD patients has been found to be significantly increased, as demonstrated (204). Furthermore, Chen et al. (133) discovered a significant increase in the ratio of Th17/Treg cells in the mesenteric lymphoid tissue of colitis rats. Conversely, the ratio of Th17/Treg cells was markedly decreased, leading to a notable amelioration of colitis after the injection of MSC-Exos via the caudal vein (180, 205). Additionally, it was found that adipose-MSC-Exos (AD-MSC-Exos) can restore the proportion of Treg cells in the spleen of the IBD mouse model to the baseline level, akin to that of normal mice, and also improve the inflammation of dextran sulfate sodium (DSS)-induced colitis (166).

### 5.4 DCs

In the intestinal mucosa, DC are the main antigen-presenting cells. They produce and secrete proinflammatory cytokines including IL-6 and IL-12, which exacerbate the inflammatory response in IBD (refer to Figure 4) (206). Additionally, their production of reactive oxygen species (ROS) contributes to the destruction of the intestinal mucosal barrier and participates in the tissue damage observed in IBD (1). The potential mechanisms through which MSC-Exos regulate DCs encompass the following: (1) MSC-Exo-treated DCs result in the downregulation of IL-4 and IL-12, coupled with the upregulation of TGF-β, thus inhibiting the maturation and differentiation of DCs (200, 207, 208). This process also induces the differentiation of T cells, affects the balance of Th1/Th2 transformation (209), and inhibits the intestinal inflammatory response (210). Furthermore, (2) MSC-Exos suppress the differentiation and maturation of DCs by modulating the TLR-NF-κB signaling pathway. In summary, MSC-Exos exert a potent immunomodulatory effect (211). In the context of IBD treatment, MSC-Exos carrying immunosuppressive factors influence M2 macrophage polarization, inhibit the proliferation of Th1 and Th17 cells, promote the differentiation of Treg cells, and induce antigen-presenting cells (212).

### 5.5 IECs

#### 5.5.1 Repair of the IEB

**Figure 6:**
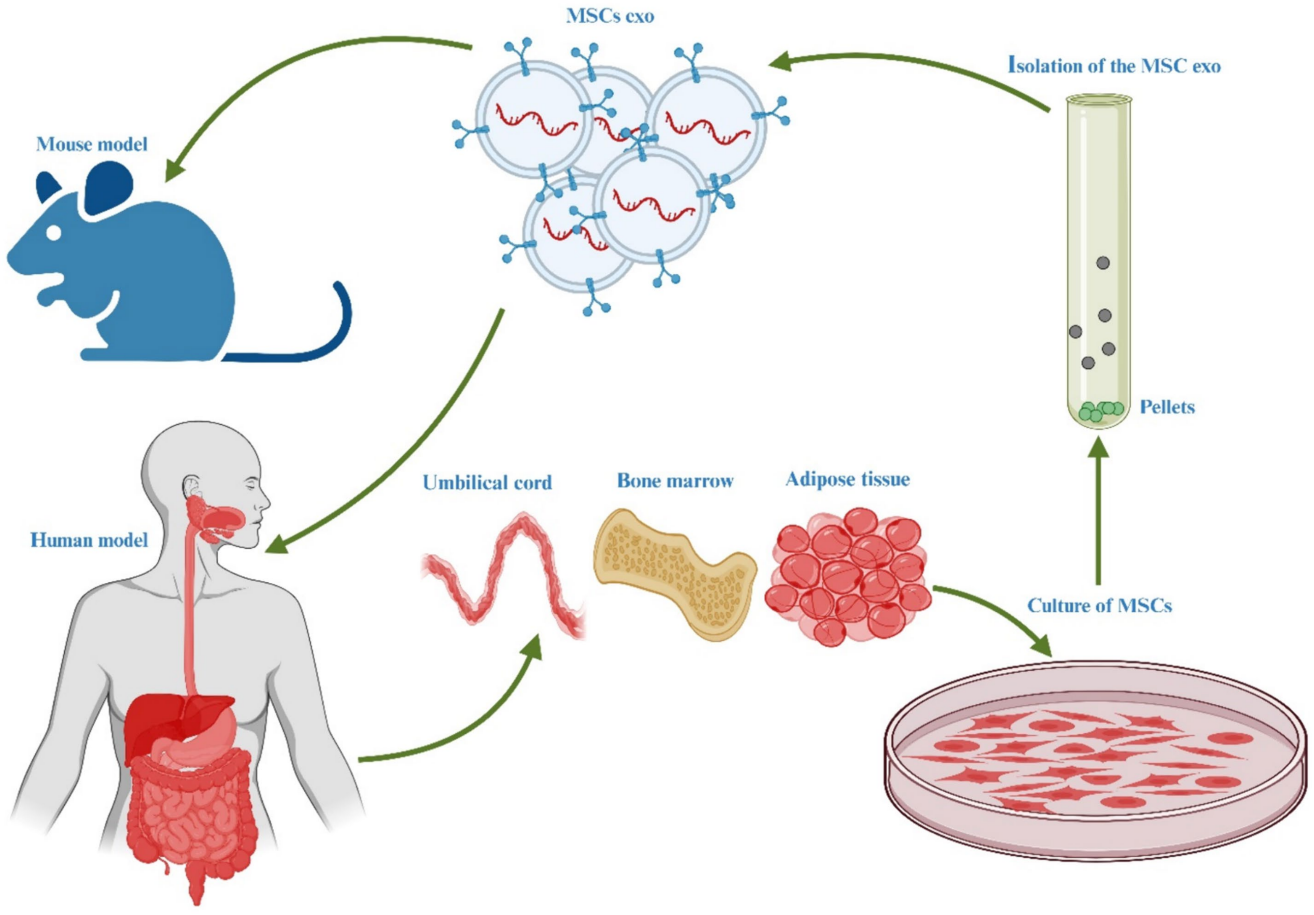
Diagrammatic illustration of the treatment of IBD by MSC-derived EVs.

The intestinal barrier encompasses the IEB, mucosal innate immune system, intestinal mucus, and intestinal microbiota. The IEB, constituted by the tight junctions of IECs, forms a crucial mechanical barrier and represents the most pivotal component of the intestinal barrier. Dysregulation and disturbances in various aspects, including mucosal immunity, surface mucus, intestinal microbiota, and oxidative stress, can compromise the integrity of the IEB, leading to the necrosis and apoptosis of IECs and an escalation in wall permeability. These changes underlie the pathological alterations observed in IBD (213–216). MSC-Exos exhibit the capacity to repair IEC injury, inhibit IEC apoptosis, preserve the equilibrium of oxidative stress, and diminish intestinal wall permeability, thereby facilitating the restoration of the IEB, refer to Figure 6.

##### 5.5.1.1 Repair the injury of IECs

Mao et al. (173) observed a significant reduction in immune-mediated damage to IECs in colitis mice at 12 h post-intravenous injection of hUC-MSC-exo, indicating the therapeutic potential of exosomes in immune regulation. Additionally, Wang et al. (47) demonstrated that ADSCs-Exos treated with vascular endothelial growth factor C (VEGF-C) upregulate miR-132 and modulate the Smad-7 gene and TGF-β/Smad signaling pathways, thereby promoting the proliferation, migration, and lymphangiogenesis of lymphatic endothelial cells (LECs). Furthermore, in a DSS-induced colitis model, hUC-MSC-exo were found to regulate the balance between ubiquitination and deubiquitination of functional proteins through miR-326, facilitating the repair of IEC damage and maintenance of IEB integrity by inhibiting neddylation (181). Although the specific regulatory process and molecular mechanism require further elucidation.

##### 5.5.1.2 Regarding the inhibition of IEC apoptosis

It has been identified in numerous apoptotic bodies in the colon mucosa of UC patients (214). Subsequent studies revealed that the lack of NF-κB in IECs is a critical factor leading to their entry into the apoptosis program (215). Yang et al. (182) observed a high expression of caspase 3, caspase 8, and caspase 9 in the cysteinyl aspartate-specific protein (caspase) family in the colon tissue of rats with colitis. Following the injection of MSC-derived EVs into the tail vein, the expressions of these caspases were altered, indicating the potential of MSC-EVs to inhibit IEC apoptosis and repair the IEB. Similarly, Yang et al. (182) obtained similar results, further affirming the ability of MSC-EVs to mitigate the apoptosis of IECs and contribute to IEB repair.

##### 5.5.1.3 Inhibition of oxidative stress

Oxidative stress plays a crucial role in the onset and progression of IBD. Typically, colonic tissue cells maintain a dynamic equilibrium between oxidation and antioxidation. However, chronic colonic inflammation disrupts this balance, leading to excessive production of reactive ROS by IEC and macrophages, which results in colonic tissue damage (217, 218). Elevated levels of ROS have been detected in the colonic mucosa of IBD patients and animal models, with a positive correlation to disease severity and increased peroxidation products, such as peroxidized lipid molecules and proteins (218–221). Overproduction of ROS directly damages nucleic acids, lipids, and proteins in colon epithelial cells, inducing necrosis or apoptosis of IECs and compromising the integrity of the IEB (222). While oxidative stress aids the immune system in pathogen clearance, excessive oxidative stress stimulates macrophages to produce proinflammatory cytokines, exacerbating intestinal inflammation. Research indicates that mesenchymal stem cell-derived exosomes (MSC-Exos) can repair various injuries caused by oxidative stress (223–225).

However, most studies have focused on tissues such as the heart, lungs, liver, and kidneys, with limited research on MSC-Exos in treating colitis (48). Nevertheless, MSC-Exos might repair oxidative stress-damaged IECs and help maintain the intestinal mucosal barrier. Additionally, MSC-Exos could mitigate oxidative stress injuries through immune regulation. Further research is needed to determine whether MSC-Exos can reduce intestinal ROS production and restore the balance between oxidation and antioxidation in the intestine.

##### 5.5.1.4 Reduction of intestinal wall permeability

It was discovered that the incidence of necrotizing enterocolitis (NEC) and intestinal wall permeability significantly decreased in NEC model rats treated with intraperitoneal injections of BM MSCs or BM MSC-Exos (226). Furthermore, McCulloh et al. (227) verified that MSC-Exos can reduce intestinal wall permeability, though the precise molecular mechanisms underlying this effect remain unclear.

#### 5.5.2 Anticolonic fibrosis

Colonic fibrosis is a chronic complication of IBD, with more than 30% of CDpatients developing fibrosis, which rapidly leads to intestinal stenosis and adversely impacts patient prognosis (228, 229). Pathological stimuli such as chronic intestinal inflammation, oxidative stress, damage to and repair of the IEB, promote epithelial-mesenchymal transition (EMT). This transition involves the loss of epithelial cell polarity and intercellular connections, morphological and functional transformation, and significant extracellular matrix (ECM) accumulation, culminating in intestinal fibrosis (230). During tissue repair or response, fibroblast activation is crucial for fibrosis development (230). The TGF-β signaling pathway has been identified as a key activator of fibroblasts, inducing their differentiation into myofibroblasts, which is central to the pathogenesis of intestinal fibrosis (231). Yang et al. (232) demonstrated that BM-MSC-Exos can significantly reverse EMT in TGF-β1-treated (IEC-6) through miR-200b, thereby mitigating intestinal fibrosis. Various studies have shown that MSCs from different sources regulate EMT by activating TGF-β and Wnt signaling pathways, reducing ECM aggregation, and exerting antifibrotic effects (233). While MSCs have been shown to reduce fibrosis in the heart, lungs, liver, and kidneys, research on their effects on intestinal fibrosis remains limited (234). Choi et al. (234) hUCMSC-Exos suppress TGF-β1-induced fibroblast activation by inhibiting the Rho/MRTF/SRF pathway, thereby reducing intestinal fibrosis. Additionally, Duan and Cao (235) discovered that human placental MSC-Exos can decrease collagen deposition in the intestinal wall by reducing collagen production and promoting its degradation through inhibition of TGF-β1 protein expression.

### 5.6 hUC-MSC-derived EVs role in other inflammatory diseases

Instead of being solely associated with IBD, hUC-MSC derived EVs have demonstrated promising potential in the treatment and management of diverse pathological conditions.

#### 5.6.1 hUC-MSC derived EVs promote functional recovery in spinal cord injury

The induction of M1 to M2 phenotypic transition in bone marrow-derived macrophages (BM-DM) can be effectively achieved through the utilization of hUC-MSC-derived exosomes, which are characterized by an average particle size of 70 nm. *In vivo* studies have provided compelling evidence that hUC-MSC-derived exosomes facilitate the process of functional recuperation subsequent to SCI through the downregulation of inflammatory cytokines, including TNF-α, MIP-1, IL-6, and IFN-γ (61). The role of hUC-MSC-exo in the transformation of macrophages from the M1 to the M2 phenotype has been established (236). Intravenous administration of these exosomes shows promise as a therapeutic approach for mitigating inflammation and facilitating the restoration of locomotor function following SCI. These findings highlight the potential of exosomes to significantly contribute to the future of SCI therapy (237).

In the treatment of SCI mice, hUC-MSC transplantation has been shown to greatly enhance the survival and regeneration of myelin and nerve cells in the injured area of the spinal cord (238). This transplantation approach also leads to a significant improvement in the motor function of the animals. These positive therapeutic outcomes may be attributed, at least in part, to the effects of hUC-MSC transplantation, which include reducing the production of IL-7 at the site of injury, enhancing the activation of M2 macrophages, and preventing inflammatory infiltration (239). A study conducted in 2022 suggests that the ability of hUC-MSCs to modulate the inflammatory response following nerve injury plays a critical role in their effectiveness in treating acute SCI. This information may guide future applications of hUC-MSCs and enhance the effectiveness of their clinical translation (240). Moreover, hUC-MSC-EVs, acting through the miR-29b-3p/PTEN/Akt/mTOR axis, have demonstrated the ability to reduce pathological alterations, enhance motor function, and promote nerve function repair in SCI rats (241).

#### 5.6.2 hUC-MSC derived EVs suppresses programmed cell death in traumatic brain injury

Globally, traumatic brain injury (TBI) is a leading cause of fatalities and long-term impairment (242). TBI treatments have recently gained significant attention. However, the therapeutic application of hUC-MSC transplantation in TBI has been limited by challenges such as immunological rejection, ethical considerations, and the potential for tumorigenicity. Notably, hUC-MSC-exo have demonstrated the ability to enhance neurological performance, reduce cerebral edema, and decrease lesion volume following TBI (243). According to a study, hUC-MSC-exo may provide neuroprotection against TBI by exerting inhibitory effects on cell death processes, including apoptosis, pyroptosis, and ferroptosis, through the PINK1/Parkin-mediated mitophagy pathway (238). Recent studies have shed light on the emerging significance of pyroptosis in the context of brain damage. It has been demonstrated that pyroptosis plays a significant role in the development of neonatal cerebral ischemia-reperfusion (I/R) injury, exerting a substantial influence on the pathological processes involved (244). hUC-MSC-exo possesses the potential to mitigate apoptosis following TBI, reduce neuroinflammation, and promote neurogenesis (245). In order to evaluate the efficacy of exosome therapy in facilitating neurological recovery, a rat model of TBI was established. Subsequent analysis revealed a significant improvement in sensorimotor function and spatial learning in rats upon administration of hUC-MSC-exo (246). Moreover, through the inhibition of the NF-kB signaling pathway, hUC-MSC-exo exhibited a substantial reduction in the synthesis of proinflammatory cytokines. Furthermore, noteworthy observations were made regarding the neuroprotective effects of hUC-MSC-exos, including the prevention of neuronal apoptosis, attenuation of inflammation, and facilitation of neuronal regeneration within the injured cortex of rats subjected to TBI (247).

#### 5.6.3 hUC-MSC derived EVs In myocardial infarction

A myocardial infarction (MI) is a significant inflammatory disorder triggered by an imbalance in substrate and oxygen supply versus demand, leading to ischemia or cellular demise (248, 249). Despite the frequent utilization of early revascularization and the widespread implementation of quality measures, notable variations exist at the local and regional levels regarding the management and outcomes of MI (250, 251). The therapeutic effectiveness of stem cell-based therapy in MI for the purposes of heart repair and regeneration has been substantiated through preclinical investigations as well as clinical trials (252). A potential therapeutic approach for MI involves the utilization of MSC-EVs. To investigate the effects of hUC-MSC-EVs loaded with miR-223 in the context of MI, experiments were conducted employing both an *in vitro* cellular model of oxidative stress and cardiac fibrosis, as well as *in vivo* rat models of MI. The transfer of miR-223 via EVs demonstrated improved cardiac function in MI rat models, along with reduced fibrosis and inflammation (253). The pathogenesis of MI is primarily attributed to the presence of inflammation, wherein the detrimental effects of MI are intricately associated with the orchestrated activation of multiple inflammatory cascades and the recruitment of inflammatory cells (254). Before the widespread implementation of cardiomyocyte regeneration in clinical trials, substantial further research and development are required. In the realm of cell therapy, extensive investigations have been conducted to explore the differentiating capabilities of (hUC-MSCs). It is widely recognized that the utilization of 5-Azacytidine (5-Aza) effectively triggers the differentiation of hUC-MSCs into cardiomyocytes, resulting in morphological changes and the expression of cardiac-specific proteins, irrespective of the presence of basic fibroblast growth factor (bFGF) (255). An additional investigation has revealed that 5-Azacytidine (5-Aza) may facilitate the *in vitro* differentiation of hUC-MSCs into cardiomyocytes through the sustained phosphorylation of extracellular signal-regulated kinase (ERK) (256). Recent scientific investigations have demonstrated that the administration of injected hUC-MSCs exerts beneficial effects on cardiac function in rats with dilated cardiomyopathy (DCM) through the attenuation of myocardial fibrosis and dysfunction. These effects are achieved by the downregulation of Transforming Growth Factor-β1 (TGF-β1) and TNF-α production (257). Furthermore, emerging scientific evidence suggests that exosomes possess potential protective properties in the context of acute myocardial infarction. These exosomes have been shown to potentially facilitate cell repair mechanisms by modulating the expression of Smad7 in cardiomyocytes (257).

#### 5.6.4 hUC-MSC derived EVs role in kidney injury repair

Depending on the concentration of serum creatinine (Scr) or glomerular filtration rate (GFR), nephrologists have classified kidney failure into two distinct syndromes: acute renal failure and chronic renal failure (258). Currently, clinical treatments for kidney injury primarily involve medication, surgery, and renal transplantation (259). As stem cell research has advanced, the preventive effects of hUC-MSCs and hUC-MSC-exo on renal tissue injury have been demonstrated (260). Acute kidney injury (AKI) refers to the clinical condition that arises from a rapid loss of renal function caused by various factors (260). Pre-renal acute kidney injury commonly arises from diminished blood volume caused by fluid loss and bleeding from various etiologies, as well as a decrease in effective arterial blood volume and alterations in intrarenal hemodynamics. Recent research has focused on the role of sepsis in causing AKI. To investigate this, a sepsis model was induced using cecal ligation and puncture (CLP), followed by treatment with hUC-MSCs (261). The data presented in the study provided evidence that the administration of hUC-MSCs resulted in substantial improvement in renal function, reduction in tissue damage, and significant enhancement of overall health in mice with sepsis (262, 263). According to the data reported, pre-treatment of hUC-MSCs with IL-1 exhibited a statistically significant augmentation in their capacity to modulate the immune system (264). In response to IL-1 stimulation, exosomes selectively encapsulated a widely recognized anti-inflammatory microRNA, MiR-146a. Subsequent delivery of exosomal MiR-146a to macrophages induced M2 polarization, leading to a reduction in kidney damage in septic mice (265). According to a study, the administration of hUC-MSCs have been demonstrated to effectively ameliorate renal ischemia-reperfusion injury (IRI) in mice. This beneficial effect is achieved through a dual mechanism involving a reduction in the infiltration of macrophages into the injured kidneys and a concomitant increase in the population of M2-like macrophages during the healing process (265). Through the modulation of inflammatory cytokine production and promotion of renal tubular cell proliferation, hUC-MSCs have been observed to expedite the recovery of renal function. Additionally, it was discovered that hUC-MSC transplantation resulted in a reduction in the levels of malondialdehyde (MDA) in renal tissues, indicating a potential protective effect of hUC-MSCs against oxidative damage and mitochondrial dysfunction in renal cells (266). Subsequent investigations revealed that the underlying mechanism responsible for cisplatin-induced nephrotoxicity was primarily attributed to the induction of oxidative stress. However, this detrimental effect can be mitigated by the administration of hUC-MSC-exo, which exert their protective effects by suppressing the activation of the p38 mitogen-activated protein kinase (MAPK) pathway (267). Renal fibrosis is a common pathway in the progression of chronic kidney disease (CKD) that often culminates in end-stage renal failure. Huang et al. provided insights into the potential therapeutic role of MSCs in mitigating obstructive chronic progressive renal interstitial fibrosis (RIF) in the context of kidney failure. Their study demonstrated that infused MSCs could effectively reach the damaged kidney tissues, offering a promising approach for addressing the underlying mechanisms involved in renal fibrosis (268).

#### 5.6.5 hUC-MSC derived EVs role in cutaneous wounds healing

Inflammatory, proliferative, and remodeling stages are just a few of the many phases that make up the complex process of cutaneous wound healing (268). Recent years have witnessed extensive exploration into benefits of direct stem cell infusion for tissue regeneration (269, 270). A degradable, dual-sensitive hydrogel incorporating exosomes extracted from hUC-MSC was synthesized. Afterwards, the *in vivo* wound healing potential, exosome identification and material properties of the hydrogel were assessed. The exosome-loaded hydrogel demonstrated substantial improvements in wound closure rates, re-epithelialization rates and collagen deposition at the wound sites, as evidenced by the *in vivo* results (269). The administration of exosomes-loaded hydrogel to the wounded sites resulted in a higher density of skin appendages, indicating a potential for achieving full skin regeneration (271).

Clinicians persistently encounter challenges in managing diabetic wounds due to the prevalence of multiple bacterial infections and oxidative damage (272). Exosomes have been widely employed as a promising nanodrug delivery strategy for the treatment of diabetic (273). To ascertain their participation in the modulation of diabetic wound healing, a co-culture of human umbilical vein endothelial cells (HU-VECs) and hUC-MSCs was established, and hUC-MSC-exo were subsequently utilized in both *in vitro* and *in vivo* experiments (274). The findings demonstrated that hUC-MSCs possess the ability to mitigate oxidative stress-induced damage to endothelial cells via exosomal mechanisms, thereby accelerating the healing process of diabetic cutaneous wounds in an *in vitro* setting (273). *In vivo* setting, it was observed that wounds treated with hUC-MSC-exo exhibited significantly accelerated re-epithelialization and elevated expression levels of CK19, PCNA, and collagen I (in contrast to collagen III) (274).

#### 5.6.6 hUC-MSC cells derived EVs role in lung injury repair

Acute lung injury (ALI) leads to the early onset of lung inflammation, capillary rupture, destruction of endothelial and epithelial cells, and breakdown of tight epithelial junctions. These factors increase alveolar epithelial permeability, causing significant pulmonary edema and, in severe cases, even death (275). In animal models, transplantation of MSCs effectively enhances the recovery from ALI (276). In the LPS-induced ALI rat model, hUC-MSCs can enhance the activity of antioxidant enzymes in lung tissue (277). Based on this, photobiomodulation (PBM) was utilized to modulate the release of pro-inflammatory chemicals, reduce their levels to a certain extent, and enhance the balance of oxidative stress at the site of injury (278). Bronchopulmonary dysplasia (BPD) is a dangerous chronic lung condition characterized by a high rate of morbidity and mortality in premature neonates (279). Transplantation of MSCs is a promising strategy for treating BPD. In a rat model of BPD, the transplantation of hUC-MSC-EVs resulted in the improvement of pulmonary hypertension, restoration of lung function, and alveolar structure (280). In a BPD model treated with hUC-MSC-EVs, the proportion of Ki-67-positive lung cells increased, indicating an upregulation of cell proliferation, whereas the proportion of TUNEL-positive lung cells decreased, suggesting a downregulation of cell apoptosis (263). Furthermore, in a hyperoxia-induced BPD model treated with hUC-MSC-EVs, there was an increase in SP-C staining, a marker of type II alveolar epithelial cells (TIIAECs), as well as CD31 staining, a marker of pulmonary vascular endothelial cells (PVECs) (281). Another study has demonstrated that intervention with PBM in hUC-MSCs naturally reduces the thickness of the alveolar septum, suppresses excessive secretion of inflammatory factors, and alleviates *in vivo* conditions of bleeding, edema, and fibrosis (278). PBM is a physical intervention that enhances the therapeutic effect of hUC-MSCs and shows promise as a therapy for the treatment of ALI. Furthermore, the use of hUC-MSCs may potentially reduce bleomycin-induced mouse mortality and attenuate lung collagen buildup (282). The transplantation of hUC-MSCs induced the proliferation of alveolar type 2 (AT2) cells, while concurrently suppressing the proliferation of lung fibroblasts (283). After the transplantation of hUC-MSCs, there is an induction of AT2 cell proliferation, concomitant with the suppression of lung fibroblast proliferation. Additionally, this transplantation leads to increased release of CXCL9 and CXCL10 by interferon-stimulated cells (IFNSMs), thereby attracting additional Treg cells to the injured lung (284). In a study, hUC-MSCs were administered, and lung morphometry was successfully enhanced (285). Further research has revealed the crucial role of exosome-resident miR-377-3p in controlling autophagy, thereby preventing LPS-induced ALI (286).

#### 5.6.7 hUC-MSC cells derived EVs role in hepatic injury repair

The liver, which is widely considered to be the most critical organ in the body, plays a pivotal role in the detoxification, secretion, and metabolism of medications and toxins (287). Acute liver failure (ALF), a pathological condition resulting from rapid deterioration in hepatic function, and is clinically characterized by jaundice, coagulopathy, and encephalopathy (288). In recent years, increasing scientific research has indicated that the transplantation of MSCs holds promise as a potential therapeutic strategy for the treatment of ALF (289, 290). Findings of ta study revealed that the administration of hUC-MSC-exo via a single tail vein injection resulted in a significant improvement in the survival rate, prevention of hepatocyte death, and enhancement of liver function in an experimental mouse model of ALF induced by APAP (291). Furthermore, the administration of hUC-MSCs effectively mitigated APAP-induced hepatocyte apoptosis by modulating oxidative stress markers (292). This was evident through the downregulation of glutathione (GSH) and superoxide dismutase (SOD) levels, as well as the attenuation of MDA production. Additionally, the excessive expression of cytochrome P450 E1 (CYP2E1) and 4-hydroxynonenal (4-HNE), which are known contributors to oxidative stress, was significantly suppressed. These findings highlight the ability of hUC-MSC-exo to regulate oxidative stress pathways, thereby conferring protection against APAP-induced hepatocyte death in the context of acute liver failure (293). In a study, mouse models of acute and chronic liver damage, as well as liver tumors, were generated by intraperitoneal injection of carbon tetrachloride (CCl4) followed by the intravenous infusion of hUC-MSC-exo. The results of the study demonstrated the hepatoprotective effect of hUC-MSC-exo, as evidenced by a significant reduction in liver damage (294). hUC-MSC-exo treatment has been shown to mitigate the expression of the NLRP3 inflammasome and associated inflammatory factors (295), offering a potential therapeutic approach for the management of acute liver failure. Furthermore, in lipopolysaccharide (LPS)-stimulated RAW 264.7 macrophages, hUC-MSC-exo demonstrated inhibitory effects on the expression of NLRP3, caspase-1, IL-1β, and IL-6. These findings highlight the immunomodulatory properties of hUC-MSC-exo, which could contribute to the attenuation of inflammatory responses in various pathological conditions (296). The proteolytic activation of pro-IL-1β is facilitated by activated caspase-1. Caspase-1, IL-1β, and NLRP3 play pivotal roles in the development of acute pancreatic and hepatic injury, inflammation, and subsequent organ damage. These factors are crucial in the pathophysiological processes associated with inflammatory responses and tissue injury in the pancreas and liver (297). The primary outcome of a recent study revealed that T-Exo treatment effectively reduces circulating levels of alanine aminotransferase (ALT), aspartate aminotransferase (AST), and proinflammatory cytokines. Additionally, T-Exo therapy attenuates the activation of proteins involved in the NLRP3 inflammation-associated pathway, thereby mitigating the pathological liver damage associated with acute ALF (298). Notably, TNF stimulation of MSCs leads to the selective packaging of anti-inflammatory microRNA-299-3p into exosomes, which can be utilized for exosomal therapy purposes (299).

#### 5.6.8 hUC-MSC cells derived EVs role in controlling blood sugar/diabetes

Diabetes mellitus (DM), a collective term referring to a group of metabolic disorders, is distinguished by the presence of elevated blood glucose levels, commonly known as hyperglycemia (300). Over the preceding half-century, the dynamics of lifestyles and the process of globalization have imparted profound ramifications on political, environmental, societal, and human behavioral domains. DM represents a complex disorder influenced by a combination of genetic predisposition and environmental factors, leading to compromised insulin secretion or insulin resistance (IR). These physiological aberrations contribute to the characteristic pathophysiology of DM and its associated metabolic dysregulation (300, 301). In recent years, obesity and physical inactivity have become more common, and rapid population expansion, aging, and urbanization have all contributed to the worldwide health issue that is DM (302). To maintain physiological equilibrium within the body’s ecosystem, it is imperative to regulate the homeostasis of blood glucose, which is contingent upon the management of vital life processes (303). In the year 2018, a team of researchers conducted an investigation into the correlation between hUC-MSC-exo and hyperglycemia induced by type 2 diabetes mellitus (T2DM) (304). The findings unequivocally indicate that hUC-MSC-exo consistently mitigated blood glucose levels in T2DM-afflicted rats subjected to a high-fat diet (HFD) and streptozotocin (STZ). Notably, hUC-MSC-exo enhanced the uptake of the fluorescent glucose analogue 2-NBDG in myotubes and hepatocytes, thereby substantiating their role in facilitating glucose absorption. Additionally, hUC-MSC-exo promoted glucose uptake in the muscles, upregulated the expression of the glucose-responsive transporter (GLUT4) in T2DM rats, and reinstated hepatic glucose homeostasis through the activation of insulin signaling. The activation of the insulin/AKT signaling pathway further corroborated the potential of hUC-MSC-exo to enhance insulin sensitivity in both *in vivo* and *in vitro* settings. Moreover, an evidence demonstrated that hUC-MSC-exo facilitated insulin secretion and islet regeneration by preventing STZ-induced caspase-3 alterations that lead to cellular apoptosis (305).

## 6 Safety and efficacy in clinical trials

Clinical trials are currently underway to evaluate the safety and efficacy of MSC-EVs in the management of IBD and its related conditions (306). Significantly, a series of phase I and II trials have been conducted, resulting in promising results. Significantly, the administration of hUC-MSC-exosomes demonstrated protective effects against weight loss, without eliciting adverse effects on liver or renal functions (307, 308). Furthermore, the versatility of hUC-MSC-exosomes was highlighted through diverse evaluations, encompassing assessments for hemolysis, activation of vascular and muscular systems, systemic anaphylaxis, pyrogenicity, and hematological markers (309). In another phase I clinical trial, four individuals exhibited a response to therapy 6 months after the initiation of treatment. Three out of the patients who underwent exosome injections achieved full recovery, while one patient reported no improvement and had active discharge from the fistula site. Furthermore, all five patients (100%) reported no occurrence of local or systemic side effects (310). In a similar vein, a team of scientists has developed a comprehensive systemic quality control system and robust testing methods to ensure the safety and efficacy of hUC-MSCs, based on a minimal set of requirements for MSC-based products. The validity of this system for quality control and assessment of hUC-MSCs as a cell-based product has been verified, as none of the qualified hUC-MSCs demonstrated any serious adverse reactions during the 1-year follow-up period, even after testing for multiple indications (311). However, to ascertain the safety and effectiveness of hUC-MSC-EVs in treating IBD and related illnesses, larger, multicenter clinical trials are necessary. Furthermore, long-term follow-up research is required to assess the durability of treatment response and the presence of any potential side effects.

### 6.1 Regulatory and manufacturing considerations

Given the variation of MSC-based therapies across countries and regions, adherence to regulations is crucial. The development of hUC-MSC-EVs as a therapeutic option for IBD and related disorders necessitates meticulous consideration of regulatory and manufacturing aspects. Considering the scalability and cost-effectiveness of production procedures is imperative to enable the widespread clinical utilization of hUC-MSCs. To successfully integrate EV-based therapies into clinical practice, it is crucial to address multiple challenges associated with scalability, standardization, and characterization of EV products. Establishing standardized manufacturing processes is essential to guarantee the reproducibility, safety, and quality of the ultimate therapeutic product (312, 313). Current efforts focus on developing strategies, such as the utilization of bioreactors and microfluidic systems, for the large-scale production of EVs (314, 315). To effectively integrate the use of hUC-MSC-EVs into clinical applications, adherence to regulatory and manufacturing factors is imperative.

The European Medicines Agency (EMA) has issued guidelines regarding the utilization of MSCs in clinical studies (316). To comply with regulatory standards, it is necessary to ensure adherence to Good Manufacturing Practices (GMP) during the production of MSC-based products. In accordance with regulatory requirements, it is mandatory to submit a Clinical Trial Application (CTA) prior to the initiation of any clinical trials.

Similarly, In the United States, the Food and Drug Administration (FDA) has established recommendations for the utilization of MSCs in clinical trials. Based on the stipulations outlined in these rules, MSCs are categorized as biological entities and, as a result, are subject to regulatory oversight by the Food and Drug Administration (FDA). In accordance with the established protocols, it is necessary to complete and submit an Investigational New Drug (IND) application prior to initiating clinical trials (317). Additionally, for the production of MSC-based products, strict adherence to Good Manufacturing Practices (GMP) is mandatory.

## 7 Conclusion

Various characteristics of hUC-MSCs, including their anti-inflammatory, immunomodulatory, and regenerative properties, have been extensively investigated both *in-vivo* and *in-vitro*. These findings suggest that hUC-MSCs hold promise for mitigating inflammation and promoting the healing of damaged tissues in individuals affected by IBD and related conditions. Consequently, hUC-MSCs may serve as a potential therapeutic intervention in the treatment of IBD and its associated ailments. The development of standardized protocols for the isolation and purification of EVs will be essential to ensure their quality, safety, and efficacy in clinical applications. Further research is needed to optimize the isolation, characterization, and administration of hUC-MSC-EVs. Furthermore, further studies are required to elucidate the molecular mechanisms and signaling pathways that underlie the therapeutic effects of hUC-MSC-EVs. This will enhance our comprehension of their mode of action and guide the development of more precise therapies. To fully realize the potential of this innovative therapy, it will be crucial to address the regulatory and manufacturing challenges as clinical trials progress. Ensuring the safety and effectiveness of hUC-MSC-EVs in human patients will be crucial, and carefully planned clinical trials will be essential in determining the therapeutic usefulness of these vesicles. Furthermore, the development of scalable manufacturing processes that can produce large quantities of high-quality hUC-MSC-EVs will be essential for meeting the demands of clinical use. There are also several challenges associated with the administration of hUC-MSC-EVs, such as determining the optimal dosage, route of administration, and frequency of treatment. Future studies should explore these parameters in order to maximize the therapeutic potential of hUC-MSC-EVs and minimize potential side effects. In addition, the development of biomarkers for patient stratification and monitoring treatment response could help to personalize and optimize hUC-MSC-EV-based therapies for individual patients.

In conclusion, hUC-MSC-EVs represent a promising and novel therapeutic approach for IBD and other related disorders. The growing body of preclinical evidence supporting their therapeutic potential, coupled with advancements in our understanding of their biogenesis, molecular mechanisms, and clinical translation, offers hope for more effective and targeted treatments for patients suffering from these debilitating conditions. By addressing the current challenges and building upon the existing knowledge, researchers and clinicians can work together to harness the full potential of hUC-MSC-EVs and bring this innovative therapy to the forefront of IBD treatment.

## Author contributions

MD: Conceptualization, Writing – original draft. AW: Conceptualization, Writing – original draft. YC: Writing – review & editing, Funding acquisition. JZ: Writing – original draft, Conceptualization. YY: Conceptualization, Writing – review & editing. ZX: Visualization, Writing – review & editing.

## Footnotes

^1^ www.exocarta.org

^2^ www.microvesicles.org
