# Corrigendum: Therapeutic role of extracellular vesicles from human umbilical cord mesenchymal stem cells and their wide therapeutic implications in inflammatory bowel disease and other inflammatory disorder

**DOI:** 10.3389/fmed.2024.1495696

**Published:** 2024-10-01

**Authors:** Muhammad Azhar Ud Din, Aijun Wan, Ying Chu, Jing Zhou, Yongmin Yan, Zhiliang Xu

**Affiliations:** ^1^Changzhou Key Laboratory of Molecular Diagnostics and Precision Cancer Medicine, Wujin Hospital Affiliated with Jiangsu University, Jiangsu University, Changzhou, China; ^2^Key Laboratory of Medical Science and Laboratory Medicine of Jiangsu Province, School of Medicine Jiangsu University, Zhenjiang, China; ^3^Zhenjiang College, Zhenjiang, China

**Keywords:** inflammatory bowel disease, human umbilical mesenchymal stem cells, exosomes, genes, extracellular vesicles

In the published article, there was an error in affiliation(s) [1, 2]. Instead of “[^1^Key Laboratory of Medical Science and Laboratory Medicine of Jiangsu Province, School of Medicine Jiangsu University, Zhenjiang, China, ^2^Changzhou Key Laboratory of Molecular Diagnostics and Precision Cancer Medicine, Wujin Hospital Affiliated with Jiangsu University (The Wujin Clinical College of Xuzhou Medical University), Changzhou, China]”, it should be “[^1^Changzhou Key Laboratory of Molecular Diagnostics and Precision Cancer Medicine, Wujin Hospital Affiliated with Jiangsu University, Jiangsu University, Changzhou, China, ^2^Key Laboratory of Medical Science and Laboratory Medicine of Jiangsu Province, School of Medicine Jiangsu University, Zhenjiang, China]”.

The authors apologize for this error and state that this does not change the scientific conclusions of the article in any way. The original article has been updated.

